# Robust Underwater Oil-Repellent Biomimetic Ceramic Surfaces: Combining the Stability and Reproducibility of Functional Structures

**DOI:** 10.1021/acsami.2c13857

**Published:** 2022-09-28

**Authors:** Ming Li, Shitong Zhou, Qingwen Guan, Weijun Li, Chang Li, Florian Bouville, Hao Bai, Eduardo Saiz

**Affiliations:** † Centre of Advanced Structural Ceramics, Department of Materials, Imperial College London, London SW7 2AZ, U.K.; ‡ School of Chemistry, University of Glasgow, Glasgow G12 8QQ, U.K.; § State Key Laboratory of Physical Chemistry of Solid Surfaces, College of Chemistry and Chemical Engineering, Xiamen University, Xiamen 361005, China; ∥ Department of Mechanical Engineering, City and Guilds Building, Imperial College London, London SW7 2AZ, U.K.; ⊥ State Key Laboratory of Chemical Engineering, College of Chemical and Biological Engineering, Zhejiang University, Hangzhou 310027, China

**Keywords:** mechanical stability, underwater low oil adhesion, underwater superoleophobic, biomimetic, ceramic, structure reproducibility

## Abstract

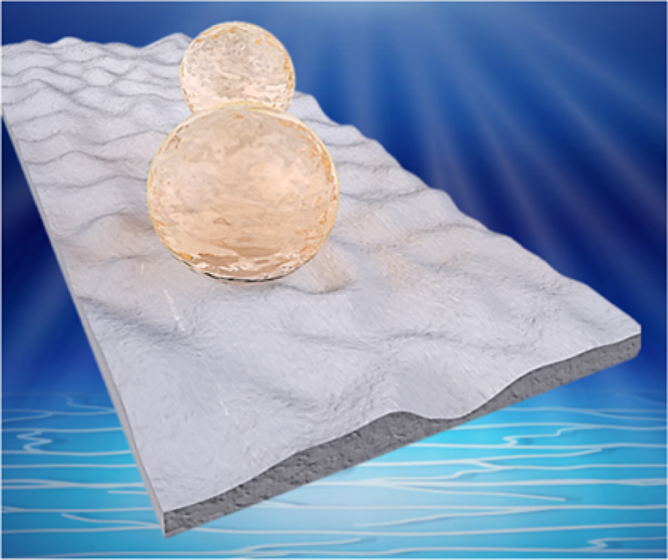

Robust underwater oil-repellent materials combining high mechanical strength and durability with superwettability and low oil adhesion are needed to build oil-repellent devices able to work in water, to manipulate droplet behavior, etc. However, combining all of these properties within a single, durable material remains a challenge. Herein, we fabricate a robust underwater oil-resistant material (Al_2_O_3_) with all of the above properties by gel casting. The micro/nanoceramic particles distributed on the surface endow the material with excellent underwater superoleophobicity (∼160°) and low oil adhesion (<4 μN). In addition, the substrate exhibits typical ceramic characteristics such as good antiacid/alkali properties, high salt resistance, and high load tolerance. These excellent properties make the material not only applicable to various liquid environments but also resistant to the impact of particles and other physical damage. More importantly, the substrate could still exhibit underwater superoleophobicity after being worn under specific conditions, as wear will create new surfaces with similar particle size distribution. This approach is easily scalable for mass production, which could open a pathway for the fabrication of practical underwater long-lasting functional interfacial materials.

## Introduction

In marine ecosystems, oil pollution caused by industrial accidental oil spills, manufacuturing, and scientific exploration has become increasingly serious.^1,2^ Meanwhile, human activity in these oil-contaminated waters is increasingly frequent. Therefore, researchers and engineers are continuously looking for new tough, superoleophobic materials exhibiting low oil adhesion underwater.^3−7^ In recent years, biomimetic surfaces with layered micro/nanoprotrusions and high surface energies have been proposed as a means to provide outstanding underwater superoleophobic performance.^8−10^ Their designs are inspired by natural examples such as shark skin,^11^ shell nacre,^12−15^ seaweed,^16^ etc. They are built with materials such as oxides,^17−19^ polyelectrolytes,^20−23^ or polymer hydrogels.^24,25^ Although these surfaces have unique superoleophobic properties in water, many of the metal oxides (i.e. CuO, ZnO) used corrode in seawater, thereby losing their properties.^17−19^ When it comes to polyelectrolytes and polymer hydrogels, neither of them has high mechanical strength, so their surface structures are easily damaged by external mechanical forces and eventually lose their excellent underwater superoleophobic performance.^20−25^ Even some underwater superoleophobic ceramic materials (i.e., TiO_2_ nanowire, cement alumina) would suffer from performance degradation when their functional surface structures are worn down.^26−28^ In the specific case of alumina, there have been reports of its use in coatings on steel meshes^27,28^ (not as a bulk material), but its adhesion to the substrate, surface structure, and chemistry may also be prone to degradation in harsh operating conditions.

To solve these limitations, researchers have developed materials with improved mechanical strength and corrosion resistance by adding inorganic nanoparticles into the polymers and building composites with surface microstructures designed to achieve underwater superoleophobicity. For example, nanoclay has been added to hydrogels to improve the mechanical strength and tensile properties of membranes,^29^ or composites that mimic the layered structure of nacre have been prepared using polyelectrolyte/clay and montmorillonite/hydroxyethyl cellulose hybrids to construct impact-resistant underwater superoleophobic films.^12,30^ Moreover, the mechanical stability of these membranes could also be enhanced by cross-linking the polyelectrolyte with inorganic nanoparticles.^21,22,31^ Unfortunately, the mechanical strength of these new functional microstructures is still limited. In summary, to fabricate durable underwater superoleophobic materials, two major goals need to be achieved: (i) the microstructure of the functional surface must have sufficient mechanical and chemical strength to resist damage and corrosion and (ii) it is necessary to design the material microstructure such that even if new surfaces are formed by wear, they retain a superoleophobic performance. However, all of the existing fabrication methods to build micro–nanostructures, whether bottom-up (photolithography, three-dimensional (3D) printing, laser deposition, layer by layer, etc.) or top-down (laser etching, chemical etching, etc.), place the functionality of the material on the surface.^7,32^ Once the structure of the surface layer wears down, the entire material loses its function. Therefore, we need to develop a new approach so that the surface structure can regenerate even if it is destroyed.

A natural example of how to combine function and regeneration can be found in the teeth distribution of sharks. Shark teeth have a hard enamel surface (Mohs hardness is about 5–8).^33^ In addition, their unique distribution effectively solves the problems caused by tooth wear or damage. Different from other animals, shark teeth are arranged in 5–6 rows (Figure S1). The outermost row contains the teeth that are actually performing the function, while the inner layers are used for replacement. Once the outermost layer of teeth is damaged and falls out, the teeth in the back row will replace them. Therefore, sharks always have hard teeth for predation. Translating this self-regenerating approach to the design of long-lasting, superoleophobic surfaces means recognizing that most surfaces can eventually get damaged and shifting to a strategy in which new surfaces are formed by the damage to retain the superoleophobic performance. Alumina ceramics have a variety of applications in tribology, wetting, health care, etc. due to their outstanding resistance to acid/alkali, salt, and wear.^6,34^ The surface morphology and bulk microstructure of polycrystalline alumina could be engineered by controlling the particle size, shape, and sintering conditions to combine high strength with superwetting properties while promoting self-regeneration.

In this study, inspired by the distribution of shark teeth and the surface structure and composition of underwater oil-resistant organisms (i.e., hydrophilic chemical composition combined with surface structures engineered at the micro/nanolevels), we built a new type of ceramic-based underwater oil-resistant substrate through gel casting and sintering. The hydrophilic properties of Al_2_O_3_ and its micro/nanoparticle sizes enable the formation of surfaces with excellent underwater superoleophobic and low oil adhesion properties. The hardness and resistance to wear and corrosion of the ceramic particles impart the surface with physical and chemical stability. As a result, it can exhibit excellent underwater oil resistance for a long period in various environments. In addition, even if the surface structure is damaged under specific conditions, the new surfaces formed will have a similar particle size distribution and retain underwater superoleophobicity. More importantly, the approach can be used to build parts with large sizes and complex shapes that could be applied to the fields of underwater antifouling surfaces and oil droplet manipulation.

## Results and Discussion

**Figure 1:**
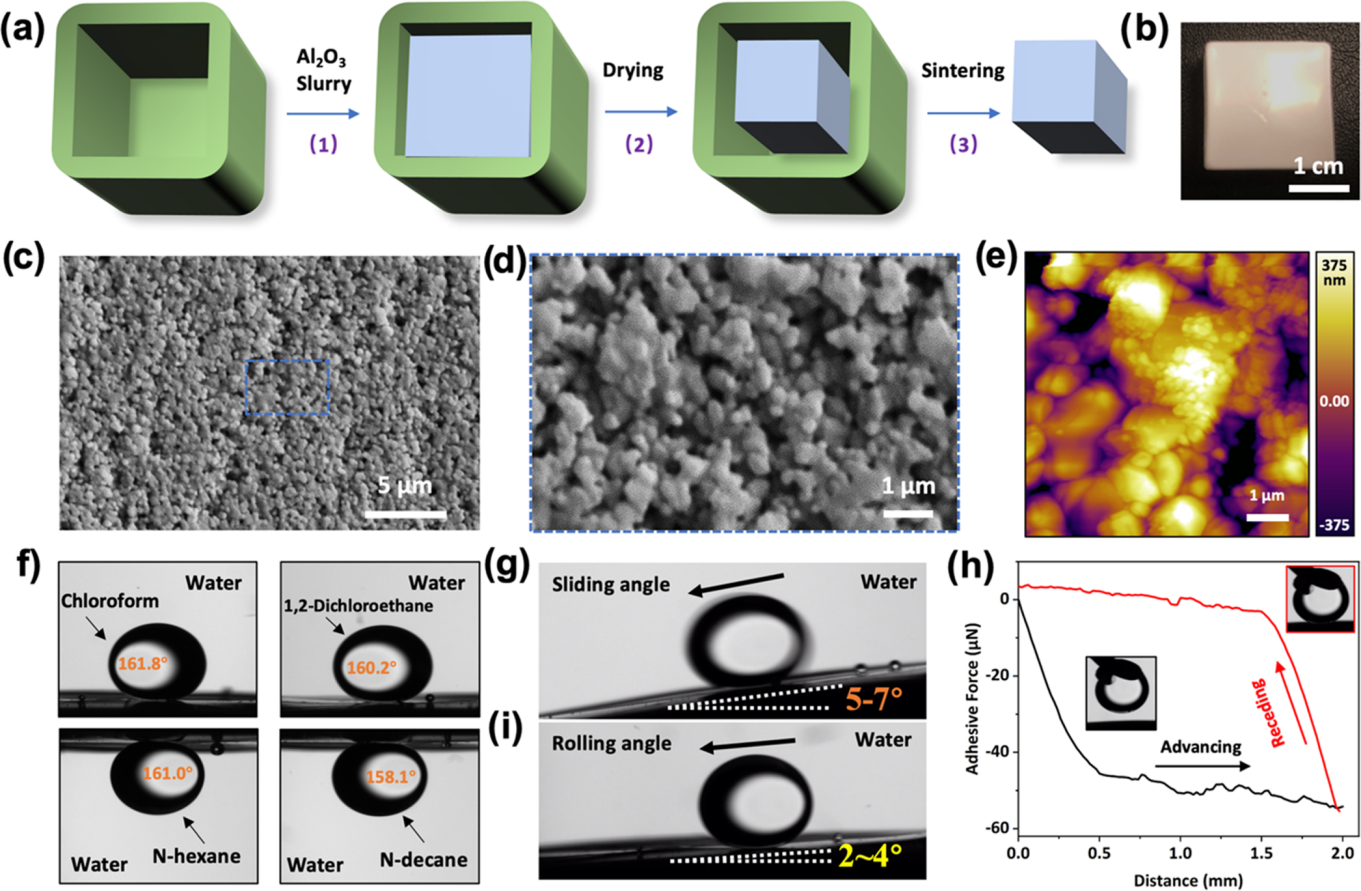
(a) Fabrication process of the Al_2_O_3_ ceramic substrate. (b) Photograph of the Al_2_O_3_ substrate using the above manufacturing method. (c, d) SEM images of the surface structure, showing the micro/nanoparticles. (e) AFM images of the surface structure, showing the roughness of the surface. (f) Static contact angles of oil droplets on Al_2_O_3_ substrate underwater; the applied oil droplets include chloroform, 1,2-dichloroethane, *N*-hexane, and *N*-decane. (g) Sliding angle of an oil droplet on Al_2_O_3_ substrate underwater; the applied oil is 1,2-dichloroethane. (h) Adhesion of an oil droplet on Al_2_O_3_ substrate underwater; the applied oil is 1,2-dichloroethane. (i) Rolling angle of an oil droplet on Al_2_O_3_ substrate underwater; the applied oil is 1,2-dichloroethane. The volume of the oil droplets used for testing is 3 μL.

In this work, we describe the design and fabrication of biomimetic, tough, and long-lasting underwater superoleophobic Al_2_O_3_ substrates (Figure 1a). The fabrication of the samples is based on Pluronic–water slurries containing alumina particles. The rheology of Pluronic gels is closely related to temperature.^35^ They exhibit low viscosity, similar to water, at low temperatures (typically below 15 °C) but form a gel at higher temperatures. We can prepare well-dispersed ceramic suspensions with high solid concentrations, use them to fill a mold when the slurry is below 15 °C, and then induce the formation of a gel able to support its own weight by increasing the temperature. Here, we cast a Pluronic-based alumina slurry, with a solid content of 70 wt %, into a silicone mold coated with a layer of silicon oil at a temperature of around 0 °C. The sample was dried at constant temperature (35 ± 1 °C) and humidity (55 ± 2%) for two weeks to obtain a cube-shaped green body (Figure S2) that was sintered at 1550 °C. The as-prepared sample (porosity: 14.5 ± 1.2%) is shown in Figure 1b, and the surface has a roughness of 0.479 ± 0.597 μm at the microscale (Figure S3) generated by the sintered micro- and nano-Al_2_O_3_ particles (Figures 1c–e and S4). This surface exhibits not only excellent underwater superoleophobicity for various oil droplets (Figure 1f and Table S1) but also low sliding angle (Figure 1g and Video S1) and low adhesion (Figures 1h and S5 and Table S1). However, in a real environment, due to the force of the water flow, the oil droplets in water tend to have kinetic energy when they contact the surface. Therefore, we not only tested the wettability of static oil droplets on the ceramic surface but also defined the rolling angle (Figure S6) to characterize the contact situation between the ceramic surface and the oil droplets with kinetic energy. Compared with static oil droplets, oil droplets with kinetic energy exhibit a lower rolling angle (Figure 1i and Video S2).

## Stability Performance of Al_2_O_3_ Substrate

**Figure 2:**
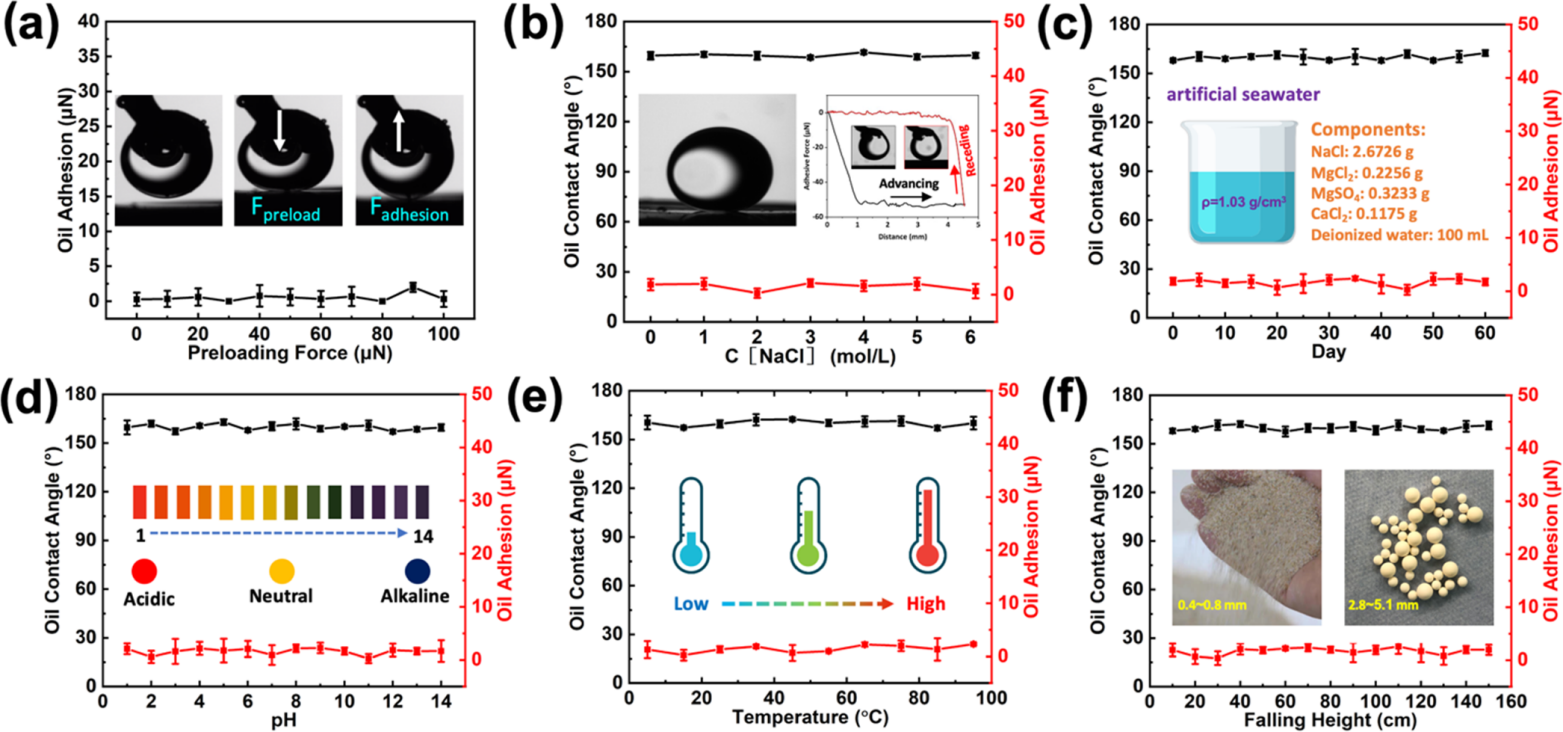
(a) Adhesive force versus preload of an oil droplet, showing stable low adhesion until the preload is up to 100 μN. The insets are representative photographs of oil droplet shapes during contacting, preloading, and detaching from the Al_2_O_3_ substrate. (b) Oil contact angle and adhesive force versus salt concentration in seawater for the Al_2_O_3_ substrate, showing stable underwater superoleophobicity and low adhesion (immersion time: 24 h). (c) Oil contact angle and adhesive force versus immersion time in seawater, showing stable underwater superoleophobicity and low adhesion. (d) Variation in the oil contact angle and adhesive force on the Al_2_O_3_ substrate after immersion in water with different pH values for 7 days. (e) Oil contact angle and adhesive force versus seawater temperature, showing stable underwater superoleophobicity and low adhesion (immersion time: 24 h). (f) Oil contact angle and adhesive force after sand grain impingement from different falling heights, showing good maintenance of underwater superoleophobicity and low adhesion. The oil used in all experiments was 1,2-dichloroethane (3 μL).

Since the oil droplets on the solid surface are under water pressure (static and dynamic pressure) in the actual marine environment,^12,13,36^ we first studied the influence of external pressure on the adhesion properties of underwater oil droplets. As shown in Figure 2a, when the external loading force increases from 0 to 100 μN, the adhesion between the oil droplet and the substrate is always close to 0, indicating that the low oil adhesion on the ceramic surface would not be affected by the external pressure (Figures S5 and S7 and Video S3).

The application prospects of underwater oil-resistant materials are highly related to their chemical stability in the working environment. In this regard, we systematically studied the chemical stability of the ceramic substrates under different seawater concentrations, immersion time, pH value, and ambient temperature (Figure 2b–e). It can be seen from Figure 2b that when the substrate is immersed in a series of NaCl solutions with concentrations ranging from 0 to saturated, the corresponding underwater oil contact angle (∼160°) and adhesion (<4 μN) remain stable. In addition, unlike previously used metal oxides (i.e., CuO, ZnO) which could not maintain their surface morphology for a long time in seawater, the Al_2_O_3_ substrate can maintain its original surface morphology even after being immersed in artificial seawater for 60 days (Figure S8), retaining underwater superoleophobicity and low oil adhesion (Figure 2c). This indicates that the material has good chemical stability in seawater and can be used for a long time in the marine environment. By monitoring the underwater oil contact angle and adhesion of a bioinspired surface after immersing in solution with different pH values for 7 days, it was found that the bioinspired surface exhibited low oil adhesion and superoleophobic properties over the whole pH range (Figure 2d). In addition, the temperature of the solution (5–95 °C) does not affect the underwater superoleophobicity and low oil adhesion properties of the ceramic surface (Figure 2e).

The mechanical stability of the microstructure is another important factor limiting the application of surfaces with special wetting. To verify the mechanical stability of the micro/nanostructure of the substrate, we used sand particles (200–800 μm) and alumina balls (2.8–5.1 mm) to simulate the impact of hard particles of different sizes and materials in seawater on the ceramic surface (Figure S9). The results showed that when the falling height of the particles increases from 5 to 100 cm, the underwater oil contact angle of the ceramic substrate remains at 160.5 ± 2.4°, and the corresponding underwater adhesion is stable at 0–3.4 μN (Figure 2f), which shows that the structure of the ceramic surface was not damaged by particle impact. The kinetic energy of the particles is significantly larger than in previously reported tests of superoleophobic surfaces in water that only used small sand particles (Table S2). In addition, we also performed finger wiping, tape peeling, soaking in freezing or boiling water, and other physical grinding methods (blade, screwdriver, steel wire ball, Figure S10) on the Al_2_O_3_ substrate to verify the mechanical damage resistance of the micro/nanostructure. The results show that the ceramic surface retained underwater superoleophobicity (∼160°) and low oil adhesion (<4 μN), which indicates that its micro/nanostructure has excellent stability (Table S3).

## Reproducibility of Functional Structures

**Figure 3:**
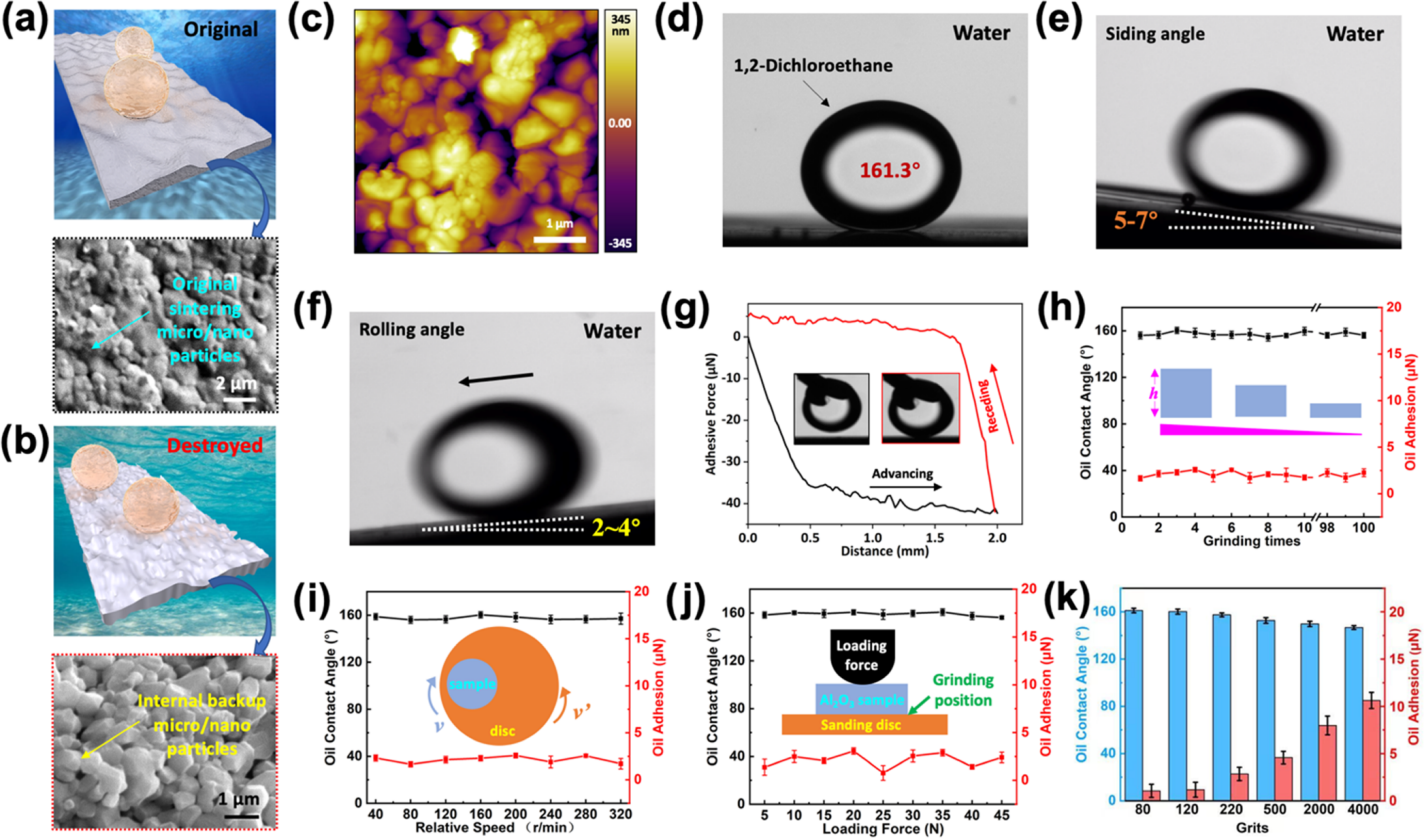
(a) Schematic diagram and SEM image of the original Al_2_O_3_ substrate surface structure. (b–h) Grinding conditions: preload of 30 N, grinding time of 5 min, and grinding speed of 100 r/min with a 120 grit diamond disc. (b) Schematic diagram and SEM images of ground Al_2_O_3_ substrate surface structure. (c) AFM images of the surface structure of the ground sample, showing the roughness after grinding. (d) Static contact angle of an oil droplet on the ground Al_2_O_3_ substrate underwater. (e) Sliding angle for an oil droplet on the ground Al_2_O_3_ substrate underwater. (f) Rolling angle of an oil droplet on the ground Al_2_O_3_ substrate underwater. (g) Adhesion of an oil droplet on the ground Al_2_O_3_ substrate underwater. (h) Relationship between the grinding time, underwater oil contact angle, and adhesive force. (i) Variation in the oil contact angle and adhesive force on Al_2_O_3_ substrates ground at different speeds. (j) Variation in the oil contact angle and adhesive force on Al_2_O_3_ substrates ground with different loading forces. (k) Variation in the oil contact angle and adhesive force on Al_2_O_3_ substrates ground using different diamond particle sizes. The oil used in all experiments was 1,2-dichloroethane (3 μL).

The strong mechanical stability of the Al_2_O_3_ ceramic substrate is mainly derived from the high hardness of alumina (∼1500 MPa, Figure 3a). Besides, its wear-rate constant is much lower than those of other materials.^37^ As a result, this substrate is more wear resistant than other underwater superoleophobic materials previously reported.^6,12,13,38,39^ Although the ceramic surface could resist physical damage under many practical situations, it could still be destroyed under more aggressive conditions (Video S4). Under these conditions, the original sintered alumina particles of the outer layer are removed, and the interior alumina particles are exposed to form a new surface (Figure S11). We explored the underwater oil resistance of the substrate after grinding away the original surface. The newly formed surfaces after grinding are shown in Figure 3b,c. They have similar microscopic roughness (0.403 ± 0.508 μm) but are flatter at the macroscale (Figure S12). Therefore, the ground Al_2_O_3_ substrates still exhibit outstanding underwater low oil adhesion and superoleophobic performance (Figure 3d–g). Moreover, the Al_2_O_3_ substrate is also highly reusable. Even after 100 times of repeated grinding, the underwater antioil performance of the substrate surface remains stable (Figure 3h).

We also investigated the influence of the relative grinding speed, the preloading grinding force, and the size of the diamond grinding particles on the underwater oleophobic performance of the ground Al_2_O_3_ surface (Figure 3i–k). The goal is to generate different degrees of physical damage. We first used a diamond grinding disc with a particle size of 137 μm to study the relationship between the underwater oil resistance of the ground surface and the grinding speed at a preload of 30 N and a grinding time of 5 min. After using different grinding speeds (40–320 r/min), the ground ceramic surfaces exhibit similar underwater oil contact angles (∼160°) and low oil adhesion (<4 μN) (Figure 3i). This is mainly because, under the same preload and grinding time and in the range of speeds used, the grinding speed only affects the thickness of the removed layer and has no significant effect on the surface morphology after grinding (Figure S13). In addition, if the relative grinding speed (130 r/min) and the grinding time (5 min) are maintained, the ceramic substrates ground with different preloads (5–45 N) also show similar underwater oil resistance performance (Figure 3j). Although the above experimental results reveal that the performance of the ground surface is stable and could withstand different types of physical damage, the underwater oil-repellent property would be affected by the grinding diamond particle size (Figure S14). As shown in Figure 3k, the underwater antioil property of the ground Al_2_O_3_ substrate would decay gradually when using grinding wheels with a grit higher than 500 (diamond particle lower than 37 μm). As the diamond particle size becomes closer to the grain size of the alumina substrate, grinding results in increasingly flat, polished surfaces (Figure S15). When we use a diamond grinding disc with large grinding particles (low grits), the ground surface roughness is still generated by grain pull-out. The surface is relatively rough and retains its underwater superoleophobic performance (Figure S16a–c). With the decrease of grinding particle diameter (increase of grits), larger, polished, flat regions emerge (Figure S16d,e). The surface is almost fully polished when using a diamond grinding disc with a particle size of 5 μm (Figure S16f). Therefore, as the size of the grinding particle decreases and the surface becomes flat (Figure S17), the adhesion of the oil droplets to the surface will increase and the contact angle will decrease. Interestingly, although the sliding angle of the ceramic surface increased as the adhesion force became larger, the rolling angle was always maintained at 2–4° (Figure S18). To prove that the reproducibility of the surface structure is achieved by the composition structure of the material, rather than grinding, we also conducted a control experiment using single-crystal alumina (sapphire). The results showed that the single crystal could not show surface structure (Figure S19), similar to our Al_2_O_3_ substrate under the same polishing conditions. This effectively proves that the reproducibility of the surface structure originates from the particle assembly structure within the substrate.

## Discussion

**Figure 4:**
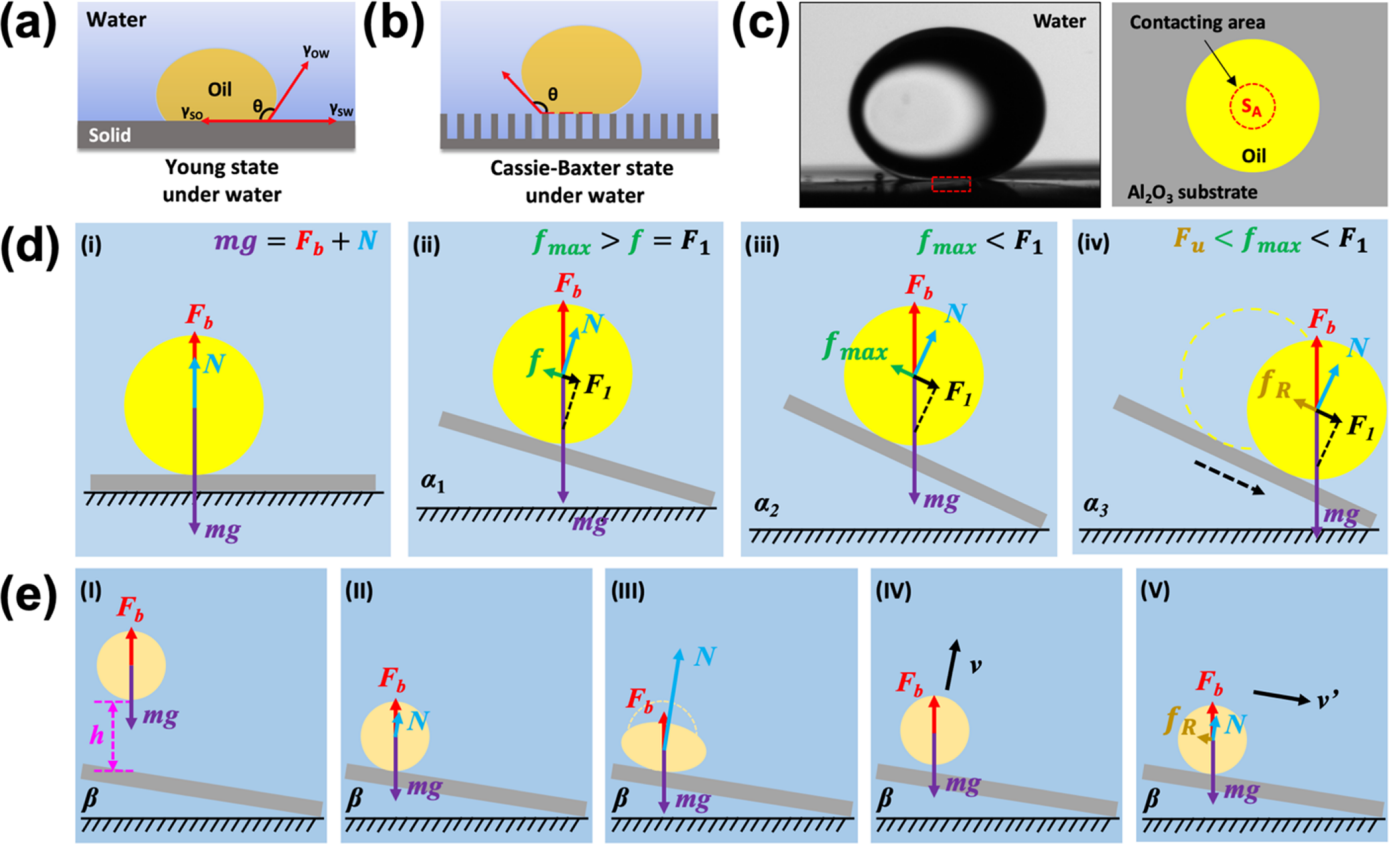
(a, b) Underwater wetting state of an oil droplet on a substrate: (a) Young’s model and (b) Cassie–Baxter’s model. (c) Contact situation of oil and Al_2_O_3_ substrate in water. (d) Schematic diagram of the underwater sliding angle test for oil droplets on the Al_2_O_3_ substrate. (e) Schematic diagram of the underwater rolling angle test for oil droplets on the Al_2_O_3_ substrate. The oil used in all experiments was 1,2-dichloroethane (3 μL).

The underwater oleophobicity of a smooth surface, which is hydrophilic and oleophilic in air, can be explained by Young’s equation describing a water/oil/solid three-phase system (Figure 4a). If θ is the contact angle between oil and solid in water^40^
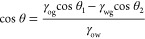
1where γ_og_, γ_wg_, and γ_ow_ are the “oil–gas”, “water–gas,” and “oil–water” interfacial tensions, respectively, and θ_1_ and θ_2_ are the contact angles between oil and solid in air and water and solid in air. Taking the “1,2-dichloroethane/water/sapphire” three-phase system as an example (sapphire is a smooth single-crystal Al_2_O_3_ surface, Figure S20), the interfacial tension of 1,2-dichloroethane in air (γ_og_) is 24.15 mN/m, and the interfacial tension of water in air (γ_wg_) is 73 mN/m.^41^ In addition, the interfacial tension between 1,2-dichloroethane and water (γ_ow_) is 28.1mN/m.^42^ In air, the contact angle of 1,2-dichloroethane on sapphire (θ_1_) is 0° (Figure S21a), and the contact angle of water on sapphire (θ_2_) is 58.6 ± 0.7° (58.4°, Figure S21b). As a result, the theoretical value of θ is 119.7 ± 1.8°, which is consistent with our experimental value of 119.2 ± 0.7° (119.1°, Figure S21c). Sapphire, which exhibits hydrophilic and lipophilic properties in air, can be oleophobic in water.

In the Al_2_O_3_ substrate, the sintered ceramic particles on the outer layer are just like micro/nanoscale protrusions (Figure 1c–e). Due to the presence of a repellent liquid phase (water), 1,2-dichloroethane only contacts the top of the ceramic particles (Figure S22). In this case, we can use Cassie’s model for the solid/oil/water three-phase system (Figure 4b), which could be expressed as follows
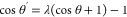
2where λ is the fraction of surface area occupied in contact with the oil droplet, θ is the contact angle of an oil droplet on an ideal smooth surface (sapphire) in water, and θ′ is the contact angle of the oil droplet on a rough surface in water.

As shown in Figure S20c, θ > 90°; thus, cos θ + 1 < 1. For a rough surface, the value of λ is always between 0 and 1(0 < λ < 1), so the value of cos θ′ is lower than 0. The smaller the value of λ, the larger the value of θ′ (Figure S23). This explains why the rough Al_2_O_3_ substrate could exhibit better underwater oleophobic properties than the polished sapphire (Figures 4c and S24). Although the surfaces of the Al_2_O_3_ substrates ground by smaller diamomd particles become increasingly flat, some microscopic roughness remains (Figure S25), so the underwater oil contact angle is still relatively large (Figures 3k and S18).

As mentioned earlier, to simulate the real application scenarios, we not only tested the contact angle, sliding angle, and adhesion of static oil droplets on the ceramic surface but also used the rolling angle to characterize the contact situation between the ceramic surface and oil droplets with kinetic energy. During the test of sliding and rolling angles, only when the resultant force of the gravity and buoyancy of the oil droplet along the inclined plane is greater than its resistance *f* in the opposite direction, the oil droplet can roll continuously on the surface (Figure 4d, *f*_max_ is the maximum static friction force before sliding). Here, we defined this resultant force along the inclined plane as the driving force (*F*_1_), and the value of this force can be expressed as
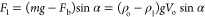
3According to the principle of force balance perpendicular to the inclined plane, we could get
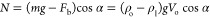
4where ρ_o_ and ρ_l_ are the densities of the oil droplet and the repellent liquid phase (water), respectively, *g* is the acceleration of gravity, *V*_o_ is the volume of the oil droplet, *F*_b_ is the buoyant force provided by the solution to an oil droplet, and *N* is the supporting force provided by the substrate. Using the inclined plane equations to calculate a friction coefficient for drop sliding (formula 3), we calculate a sliding friction force (*f*_max_, 1.5 ± 0.2 μN) that is much larger than the value for rolling (*f*_R_, 0.7 ± 0.2 μN), so the state of the oil droplet changed from sliding to rolling (Figure 4d(iv)). That is the reason why we saw the oil droplets rolling down when we tested the sliding angle (Video S1).

We can additionally use a setup in which the oil droplets already have kinetic energy when they contact the surface by increasing *h* (Figure 4e(I),(II)). Part of the kinetic energy of the oil droplet could be dissipated and the other part transformed into deformation potential energy for the oil droplets under the action of the supporting force (*N*) after they collide with the surface (Figure 4e(II),(III)). The deformation potential energy would be further transformed into the energy required to overcome adhesion and the kinetic energy of the oil droplet that bounces and leaves the surface (Figure 4e(IV)). When the oil droplet falls on the ceramic surface again, it already has a partial velocity along the inclined plane and will keep moving on the substrate (Figure S26). This movement could inhibit the replacement of water with oil at the drop–substrate interface, thereby reducing the actual contact area (*S*_A_′) between the droplets and the surface (Figure S27). Therefore, rolling can be achieved at a small inclination angle (Video S2). For oil droplets with large kinetic energy, the process of Figure 4e(II),(III),(IV) could repeat during rolling until the excess energy is dissipated (Video S5). Compared to the sliding test, the driving force (*F*_1_) of the oil droplets in rolling tests only needs to overcome the corresponding rolling friction force (*f*_R_) to achieve continuous rolling (Figure 4e(v)). This explains why the rolling angle of the oil droplets in this work is smaller than the sliding angle (Figure 1g,i).

## Conclusions

In summary, we developed a new strategy to build durable underwater oil-resistant Al_2_O_3_ surfaces with superior underwater antioil properties. Due to the outstanding wear resistance, salt tolerance, and antiacid/alkali properties of alumina, the surface structure at the micro–nanolevel could exhibit excellent chemical and mechanical stability in seawater and other solutions under extreme conditions, such as strong acidity, strong alkalinity, high salt concentration. Therefore, this surface shows long-lasting underwater low oil adhesion and superoleophobicity in complex environments. In addition, because the microstructure (in particular the grain size distribution) of the material is homogeneous, as the surface wears down, the new surfaces formed have a similar nano- to microstructure and will retain the superoloephobic behavior. Based on the excellent superoleophobicity in water and abrasion resistance of the material, as well as the conformability of the sample shape, we believe that it can be used in the manufacture of tiles and plates to coat vehicles and tools for underwater exploration, such as submarines, deep sea exploration boats, etc. The results can also guide the development of durable superoleophobic microstructures in coatings and bulk materials using other fabrication methods. Our approach does not rely on the use of fine microprocessing technologies that usually result in very delicate surfaces with limited useful life. Often the answer to this problem has been the search for highly durable surfaces that will remain unchanged under all kinds of conditions with all of the difficulties that this entails. Our work suggests that, following natural examples, a path to long and durable function may be to create materials whose surface regenerates during use while retaining its structure.

## Experimental Section

### Materials

Alumina powders (diameter: 200–300 nm) were purchased from Baikowski. Dolapix was purchased from Zschimmer & Schwarz. Pluronic 127, octanol, 1,2-dichloroethane, trichloromethane, n-decane, n-hexane, sapphire, and other reagents, including NaCl, CaCl_2_, MgCl_2_, MgSO_4_, were of analytical reagent grade and obtained from Sigma-Aldrich. All reagents were used directly and did not require further purification.

### Preparation of Al_2_O_3_ Slurry

As-received alumina particles from the supplier were first put in a vibration screening machine and vibrated for 30 min at an amplitude of level 3 to achieve initial dispersion of the agglomerated particles. Such deagglomerated particles were used to prepare ceramic slurries. Next, the Al_2_O_3_ powders were mixed with a Pluronic aqueous solution (25 wt %); Dolapix was also added to disperse the agglomerated particles and form ceramic slurries (the mass ratio of each component in the Al_2_O_3_ slurry is as follows: Al_2_O_3_ powder:Pluronic solution:Dolapix = 70:29:1). Then, 3–5 drops (5–10 μL) of octanol were added to degas the slurry and avoid air bubble voids in the slip-cast ceramics. Followed by 4–6 rounds of mixing in a Thinky ARE-250 planetary mixer for 2 min at 2000 r/min, ice water was used to cool the container after each round of mixing. Finally, the slurry is defoamed in the planetary mixer for 10 min at 2200 r/min.

### Drying and Sintering

The slurry was slip-cast in a low-permeable silicone open mold, which is covered with a layer of silicone oil on the inside, to form cuboidal-shaped green compacts. The silicon molds filled with the ceramic slurry were dried in a constant-temperature and -humidity oven at a temperature of 35 ± 1 °C and relative humidity of 55 ± 2% for 2 weeks. The green compacts were first heated to 350 °C at a heating rate of 1 K/min with a 1 h isothermal stage at 350 °C to remove the binder (Pluronic), dispersant (Dolapix), and surfactant (octanol). Then, the samples were heated to 500 °C at a heating rate of 2 K/min with a 2 h isothermal stage at 500 °C to remove further the remaining organic matter. Next, the samples were presintered to 600 °C at a heating rate of 5 K/min with the 2 h isothermal stage at 600 °C. Finally, the presintered samples were sintered at 1550 °C at a heating rate of 10 K/min with the 2 h isothermal stage at the highest temperature.

### Characterization

Scanning electron microscopy (SEM) images were taken with a JEOL JSM-6010LA scanning electron microscope. Photographs were taken with an SLR camera (Canon-EOS-760D). Atomic force microscopy (AFM) images were captured on a Bruker Dimension Icon with Scan ASYST. Oil contact angles and sliding angles were measured on an OCA 20 machine (Data Physics, Germany) in a water/air environment. The adhesion force was measured using a high-sensitivity microelectronic mechanical balance system (Data Physics DCAT 11, Germany) in a water environment. The porosity was measured based on the Archimedes drainage method. The surface roughness was tested by a Zygo NewView 200 3D optical interferometer. The hardness tests were performed using a macrohardness machine (Indentec) with a load time of 10 s, a magnification of 10×, and a load of 1 kg.

## Supporting Information Available

Description of various testing methodologies, characterizations, experimental mechanisms, and explanation of this material (Tables S1–S3, Figures S1–S27) (PDF)
Sliding angle of oil droplet on Al_2_O_3_ substrate under water (MP4)
Rolling angle of oil droplet on Al_2_O_3_ substrate under water (MP4)
Stretching deformation situation of the oil droplet during the separation of the oil droplet from the sample with different preloading force (MP4)
Alumina substrate will be destroyed when it is polished by zirconia material (MP4)
Rolling angle of oil droplet with high kinetic energy on Al_2_O_3_ substrate under water (MP4)
