# Supplementary material for: Robust Underwater Oil-Repellent Biomimetic Ceramic Surfaces: Combining the Stability and Reproducibility of Functional Structures
Source: ACS Appl Mater Interfaces. 2022 Sep 28;14(40):46077–85. doi: 10.1021/acsami.2c13857 (PMC9562273; doi:10.1021/acsami.2c13857)
Supplement: Supplementary file 1 — am2c13857_si_001.pdf [file am2c13857_si_001.pdf]

Supporting Information

**Robust Underwater Oil-repellent Biomimetic Ceramic Surfaces: Combining the Stability and Reproducibility of Functional Structures**

*Ming Li [a,*], Shitong Zhou [a], Qingwen Guan [b], Weijun Li [c,*], Chang Li [d], Florian Bouville [a], Hao Bai [e], Eduardo Saiz [a,*]*

[a] Centre of Advanced Structural Ceramics, Department of Materials, Imperial College London, London, SW7 2AZ, UK. Email: m.li19@imperial.ac.uk; e.saiz@imperial.ac.uk

[b] School of Chemistry, University of Glasgow, Glasgow, G12 8QQ, UK

[c] State Key Laboratory of Physical Chemistry of Solid Surfaces, College of Chemistry and Chemical Engineering, Xiamen University, Xiamen 361005, China. Email: lwjdesky@163.com

[d] Department of Mechanical Engineering, City and Guilds Building, Imperial College London, London SW7 2AZ, UK

[e] State Key Laboratory of Chemical Engineering, College of Chemical and Biological Engineering, Zhejiang University, Hangzhou, 310027, China

**Supporting Video 1:** Sliding angle of oil droplet on $Al_2O_3$ substrate under water.

**Supporting Video 2:** Rolling angle of oil droplet on $Al_2O_3$ substrate under water.

**Supporting Video 3:** The stretching deformation situation of the oil droplet during the separation of the oil droplet from the sample with different preloading force.

**Supporting Video 4:** The alumina substrate will be destroyed when it is polished by zirconia material.

**Supporting Video 5:** Rolling angle of oil droplet with high kinetic energy on $Al_2O_3$ substrate under water.

**Table S1.** Underwater oil contact angles and adhesive forces of $Al_2O_3$ composite materials.

| Oil Type | Contact Angle (°) | Adhesive Force (μN) |
|---|---|---|
| 1,2-dichloroethane | 158.6±1.8 | 1.4±0.5 |
| trichloromethane | 160.1±1.9 | 3.0±0.7 |
| n-decane | 159.7±3.7 | 2.1±1.0 |
| n-hexane | 161.4±2.7 | 1.9±0.9 |

**Table S2.** Mechanical properties of underwater oil-repellent materials from the references.

| Sample | Particles Used | Maximum Test Falling Height (cm) | Reference |
|---|---|---|---|
| MMT-HEC | Sand grains with diameters of 200–600 μm | 52 | [1] |
| PAA/PVDF–GN | 5 g of sand grains | 50 | [2] |
| ADP coated copper micro/nanostructure | Sand grains with sizes ranging from 400 μm to 800 μm | 70 | [3] |
| Nacre-inspired mineralized (NIM) film | Sand grains | 60 | [4] |
| $Al_2O_3$ substrate | Sand grains (200-800 μm) and alumina balls (2.8-5.1 mm) | 100 | This work |

**Table S3.** Underwater oil contact angles and adhesive forces after different physical treatments.

| Tests | Contact Angle (°) | Adhesive Force (μN) |
|---|---|---|
| Finger wipe | 158.2±1.6 | 2.7±1.4 |
| Tape peel[a)] | 159.9±4.3 | 1.9±1.4 |
| Freezing water[b)] | 161.5±3.2 | 2.9±1.0 |
| Boiling water[c)] | 159.9±3.6 | 1.6±1.0 |
| Sand grain impact[d)] | 160.9±4.7 | 1.3±0.7 |
| Blade scraping | 158.8±1.8 | 2.7±1.2 |
| Screwdriver scraping | 160.1±1.9 | 1.6±1.1 |
| Steel wire ball friction | 160.9±4.4 | 3.0±0.6 |

[a)] A strip of adhesive tape was glued to the surface of the sample under a pressure of ≈500 kPa and then manually peeled from the surface of the sample;
[b)] The sample was put in 0 ºC water for 3 h;
[c)] The sample was put in 100 ºC water for 3 h;
[d)] 5 g of sand grains impacted from the height of 100 cm.

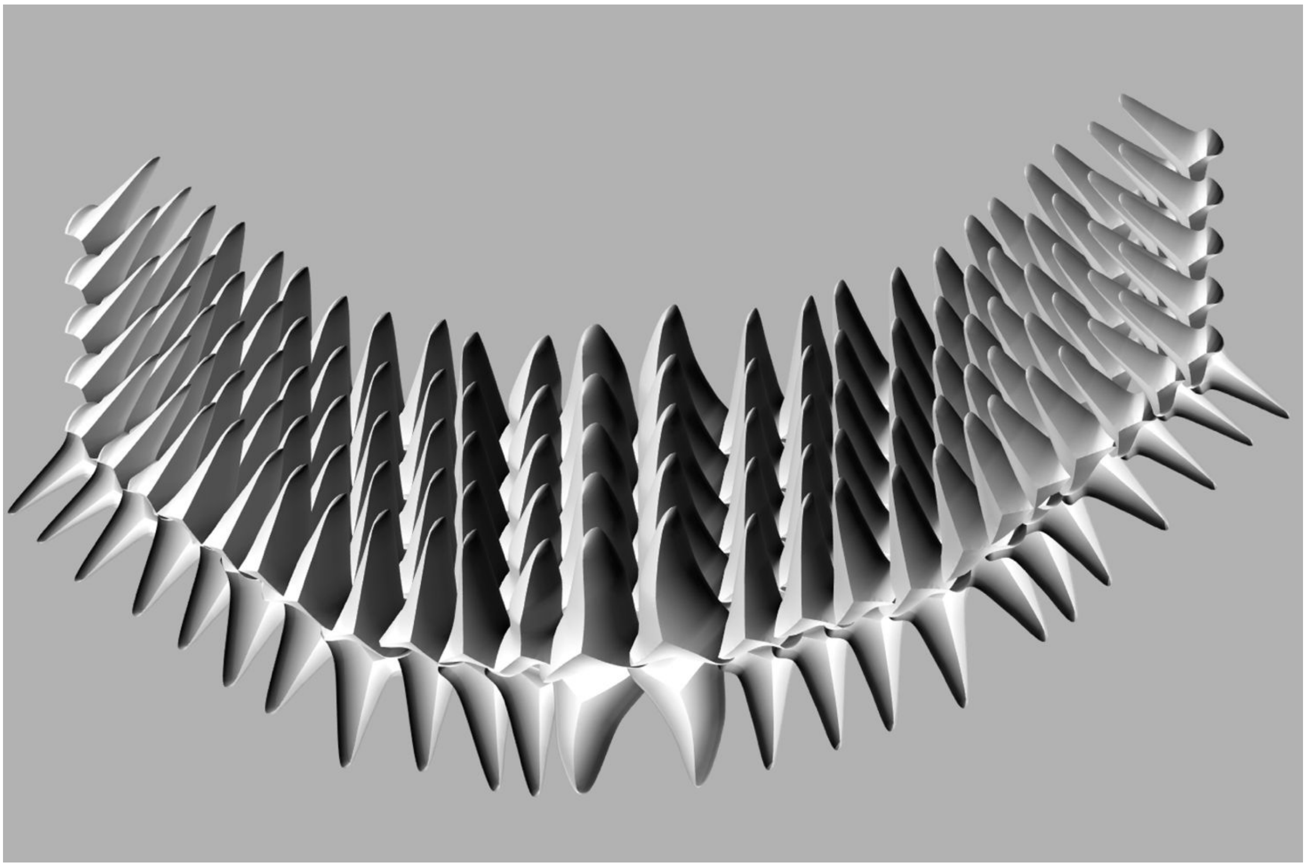

**Figure S1.** Skematics of the distribution of shark teeth, which shows the arrangement of 5-6 rows.

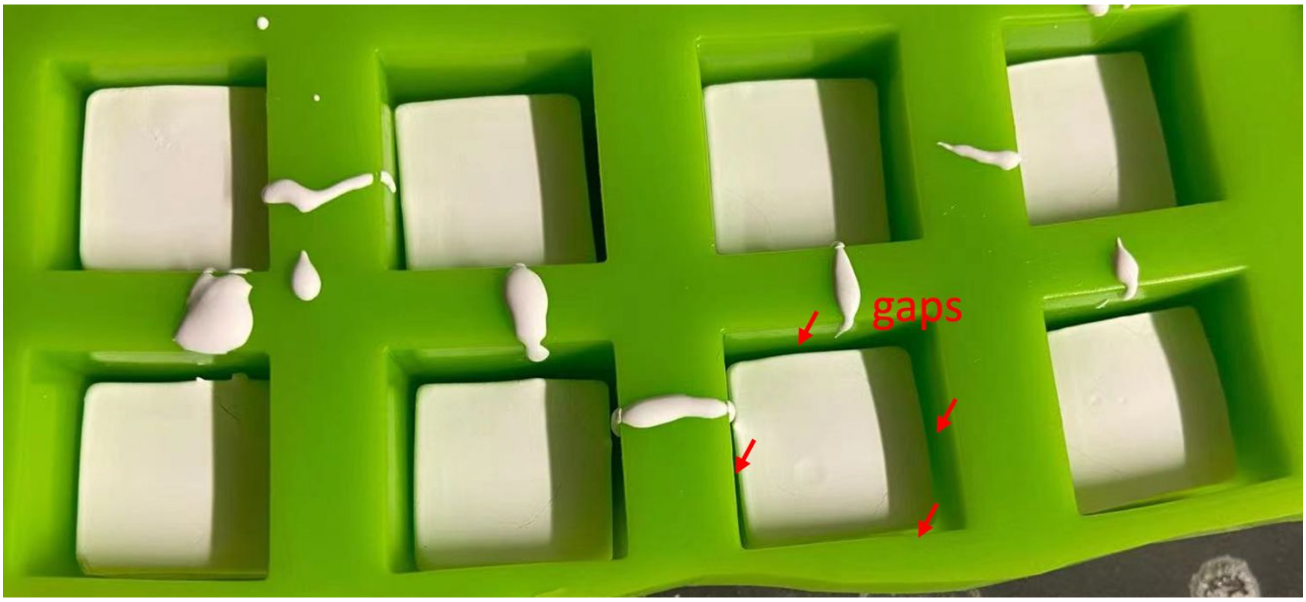


**Figure S2.** Photograph of the $Al_2O_3$ green body after drying, showing a gap between the green body and the silicon mold formed.

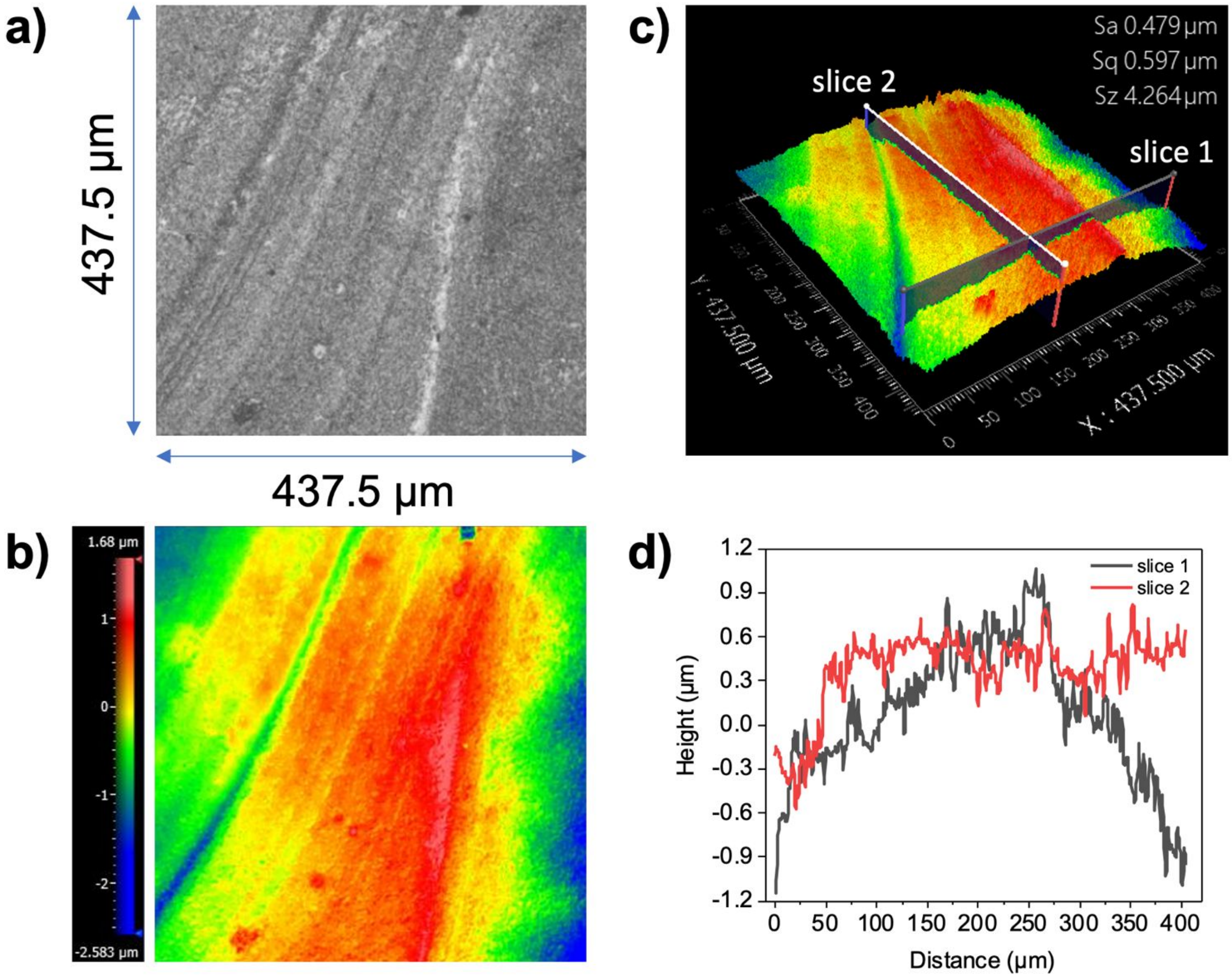


**Figure S3.** a) Zygo optical image of the location for surface topography analysis. b) Zygo 2-dimensional surface topography image of the location. c) Zygo 3-dimensional surface topography image of the location. d) The change of the surface topography in the target area along the slice.

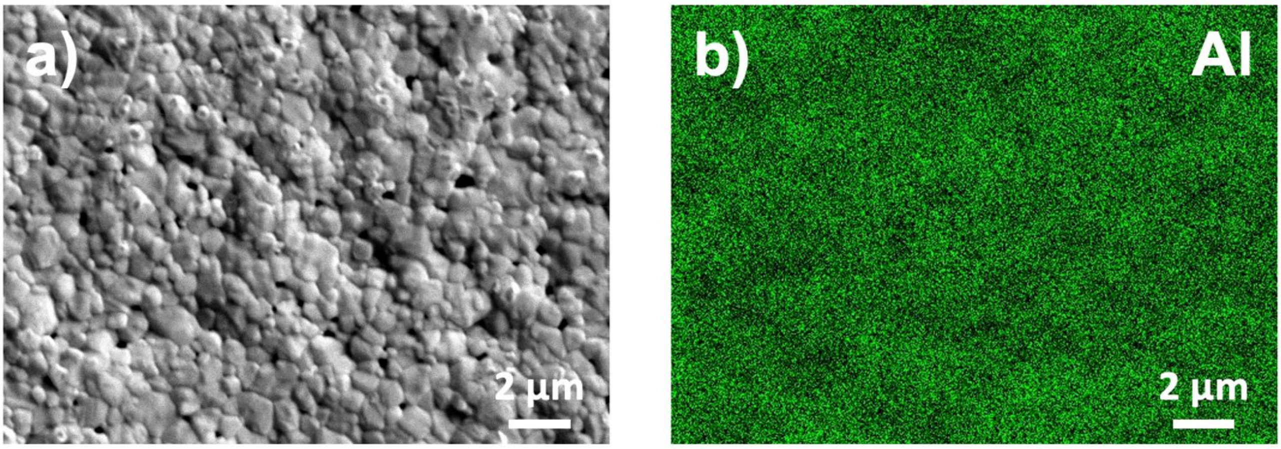

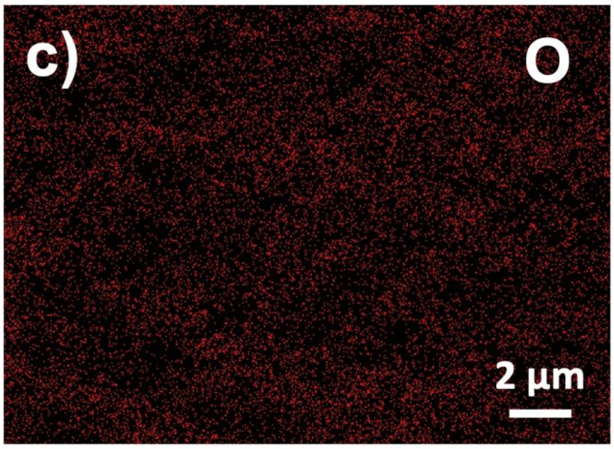


d)

| Element | ms % | mol % |
| --- | --- | --- |
| Al | 74.21 | 63.05 |
| O | 25.79 | 36.95 |
| Total | 100 | 100 |

Figure S4. a) SEM image of $Al_2O_3$ substrate. b-c) Distribution image of elements. b) element Al. c) element O. d) element percentage.

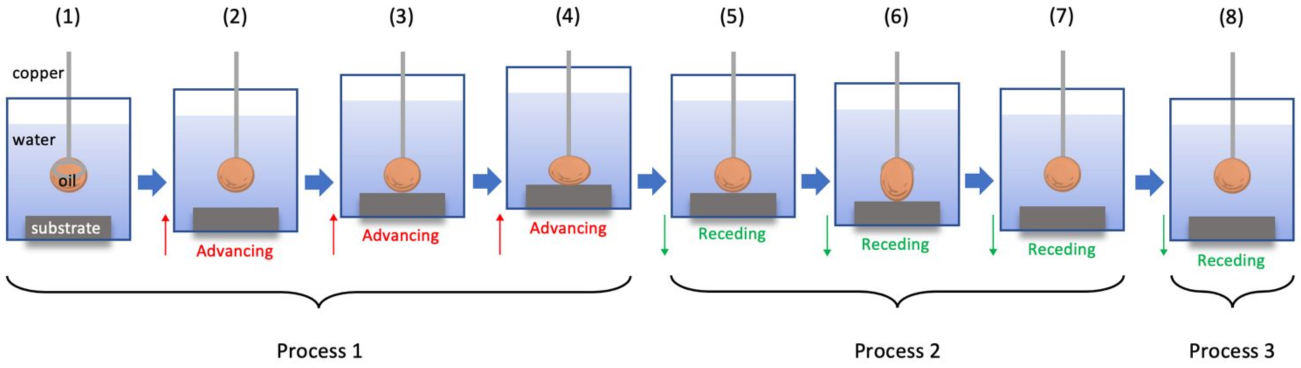


**Figure S5.** The process of underwater oil droplet adhesion test. In the process 1, the substrate is slowly approaching to the oil droplet in the steps from 1 to 3, and contact with the oil droplet at step 3. Step 4 refers the oil droplet suffer from external pressure when contact with the substrate. In process 2, the substrate is moving downwards in the steps from 5-7, and if there is adhesion between oil droplet and substrate, the shape of oil droplet would change (step 6). In process 3, the substrate goes back to its original position.

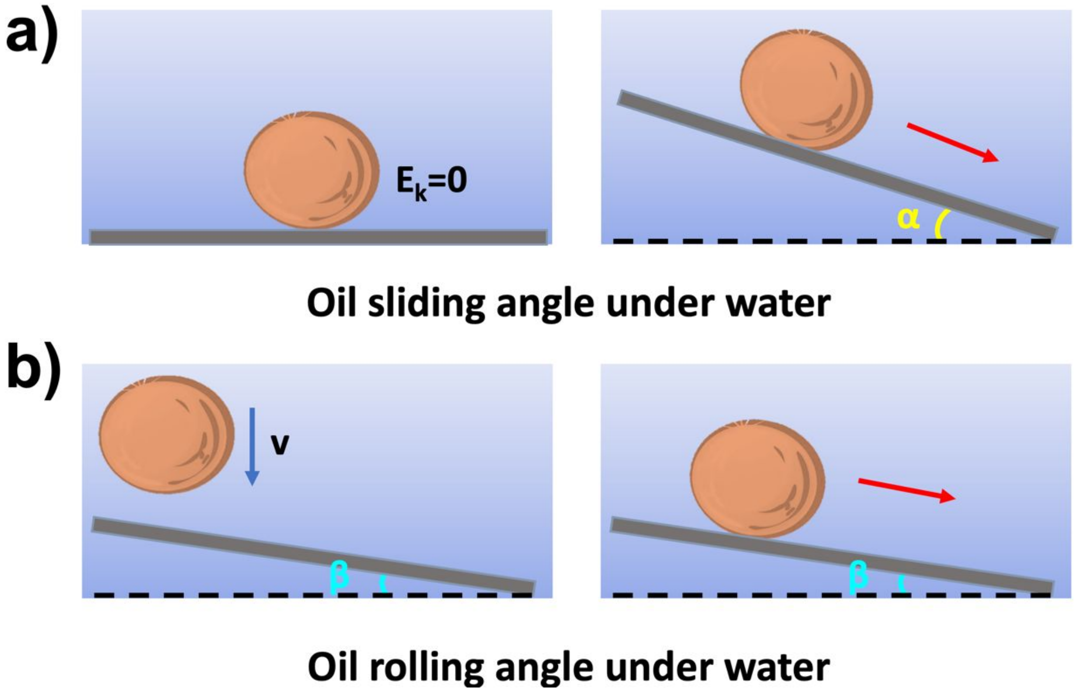


**Figure S6.** a) Schematic diagram of underwater oil sliding angle test on $Al_2O_3$ substrate. b) Schematic diagram of underwater oil rolling angle test on $Al_2O_3$ substrate.

**Supplementary explanation:**

In this work, the oil sliding angle refers to the minimum inclination angle of the ceramic surface that can keep the originally static oil droplets moving on the surface. However, the oil rolling angle refers to the minimum inclination angle at which the ceramic surface can keep the oil droplets with kinetic energy moving on the surface.

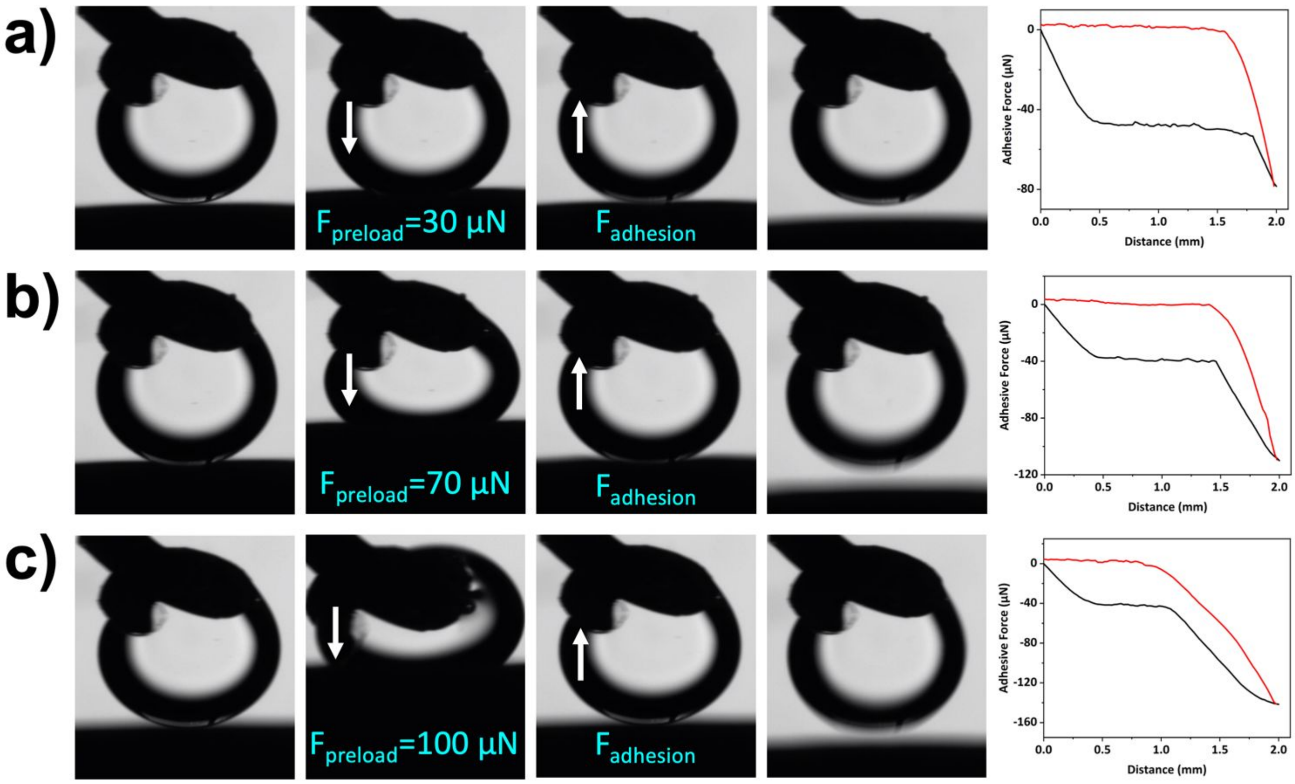


**Figure S7.** Photographs and force-distance curves during dynamic oil adhesion measurements with different preloading forces on the oil droplet: a) 30, b) 70, and c) 100 μN. The surface exhibits an ultralow oil adhesion until the preload reaches 100 μN, where stretching deformation of the oil droplet does not occur during the separation of the oil droplet from the sample. The applied oil was 1,2-dichloroethane (3 μL).

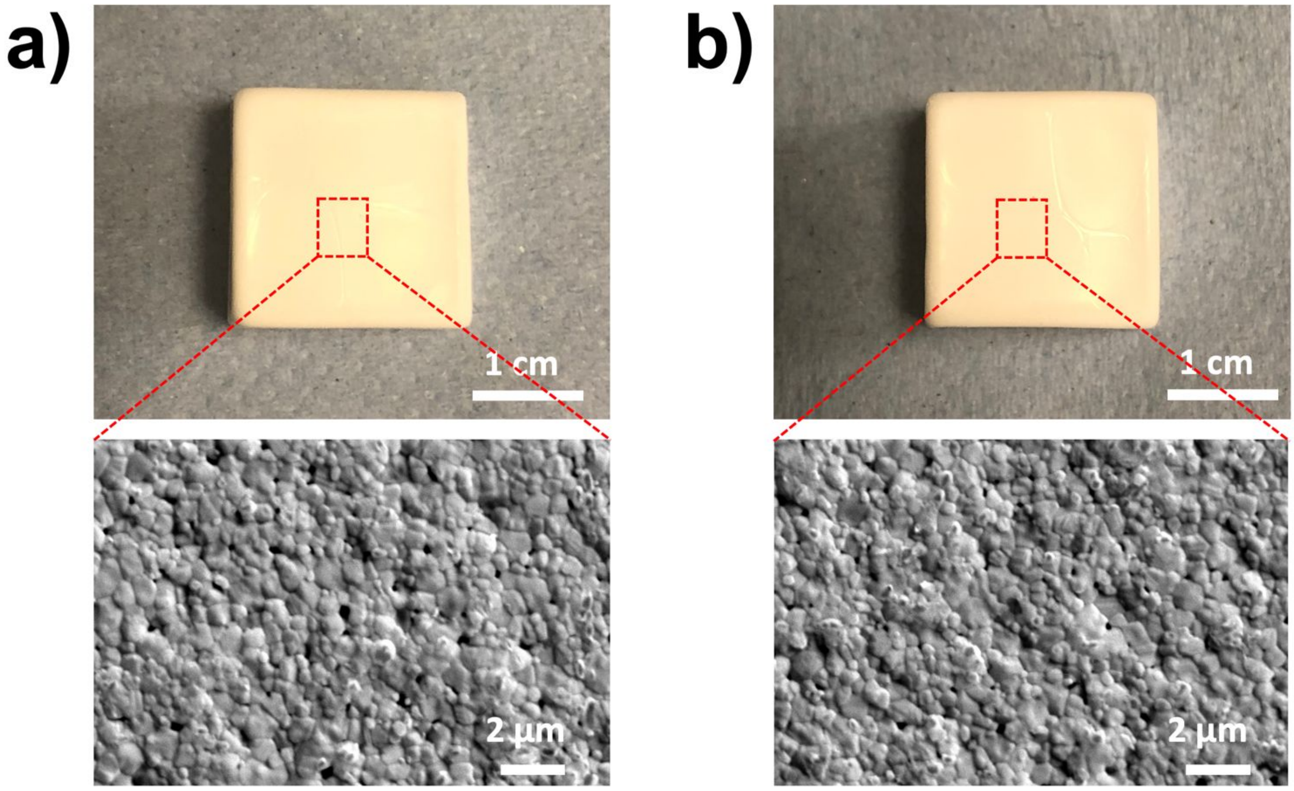


**Figure S8.** a) Photographs and SEM images of the sample's surface structure before being immersed in NaCl solutions. b) Photographs and SEM images of the sample's surface structure after being immersed in artificial seawater for 60 days.

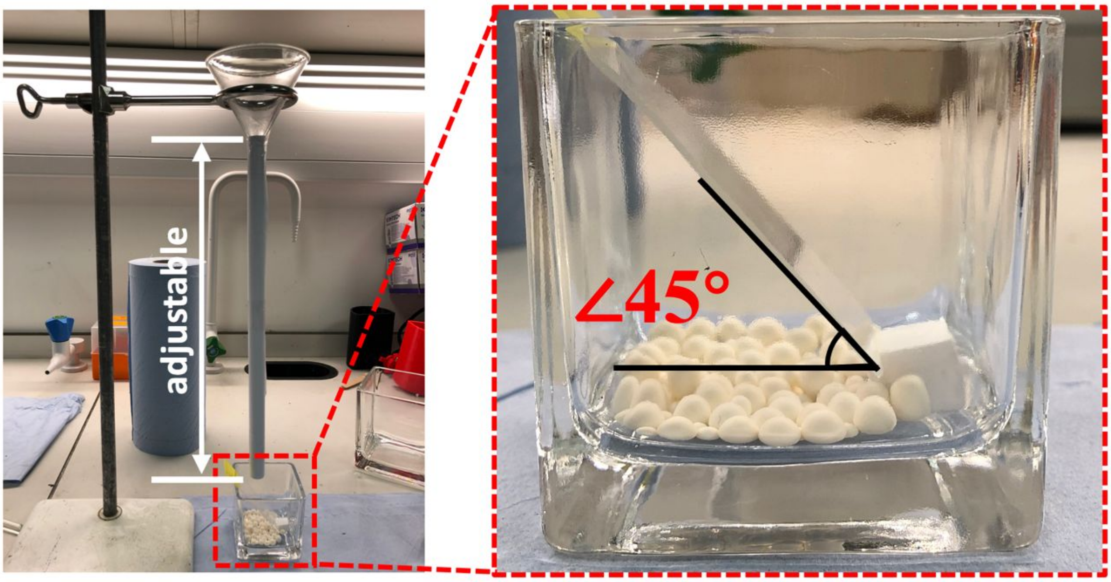


**Figure S9.** Setup for the particle impingement measurement.

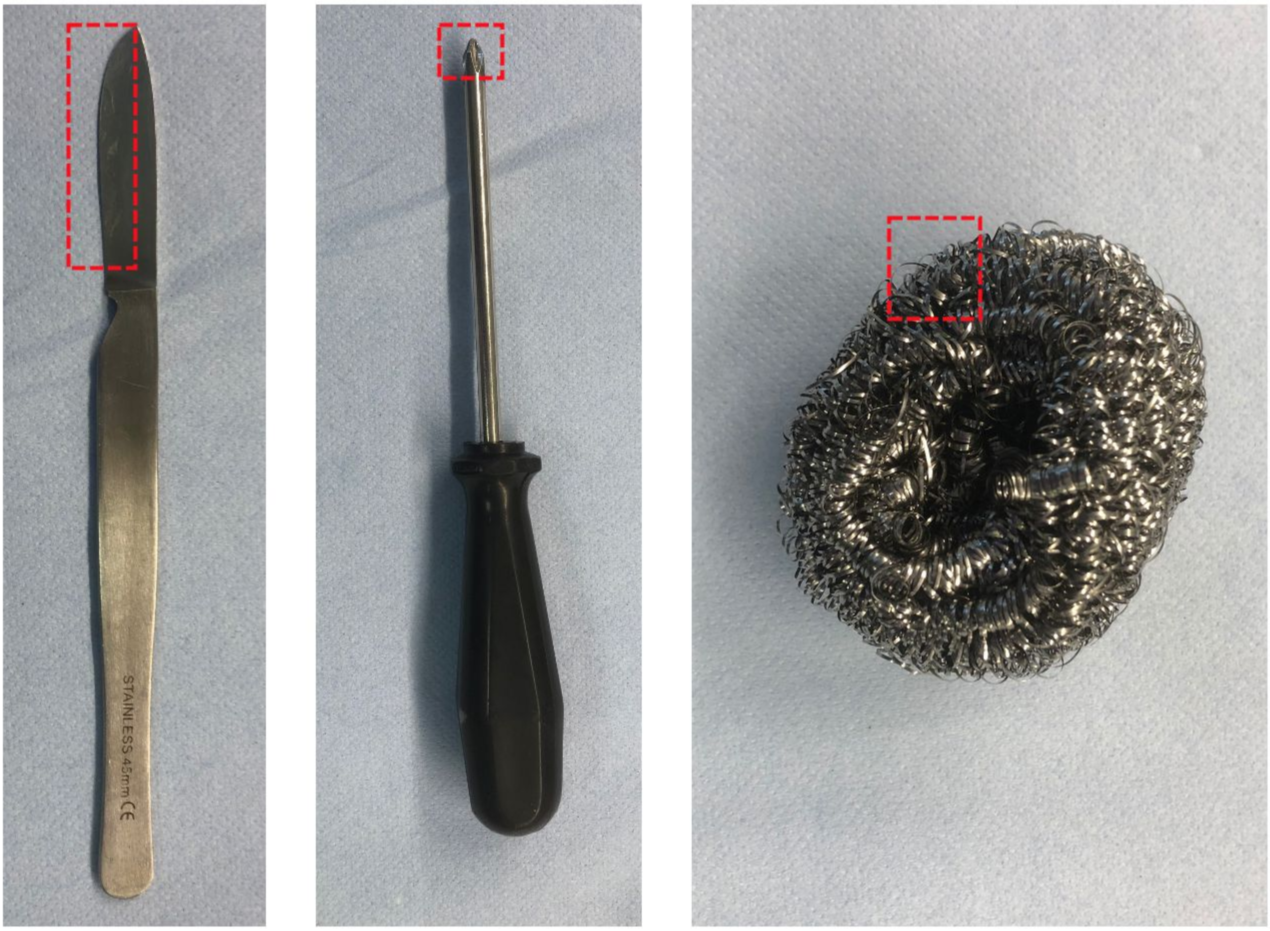


**Figure S10.** Photographs of the blade, screwdriver, steel wire ball that are used for destroying the $Al_2O_3$ substrate, the position circled by the red dashed frame is the part for contact with the $Al_2O_3$ substrate.

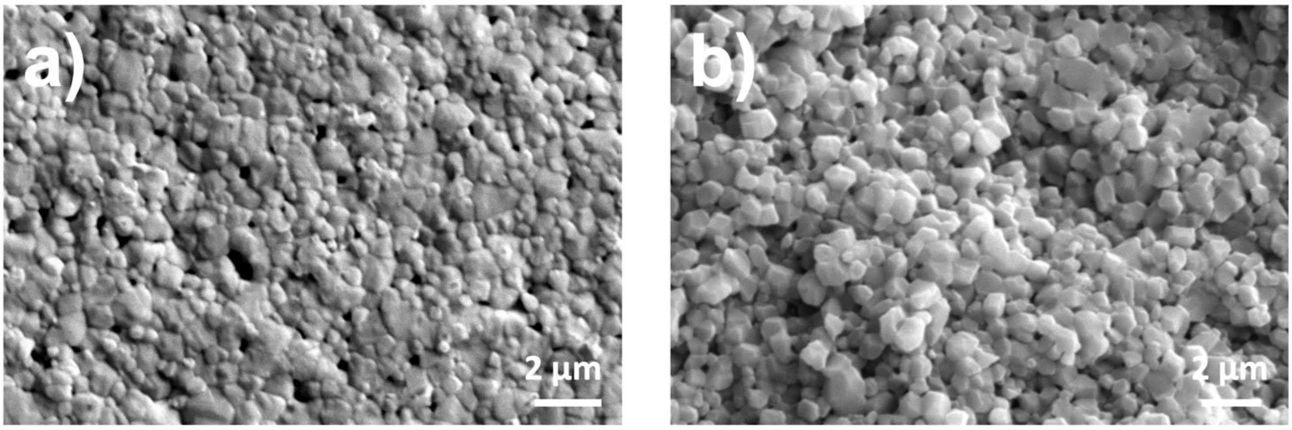


**Figure S11.** a) SEM image of the outermost sintering alumina particles. b) SEM image of the backup (internal) alumina particles.

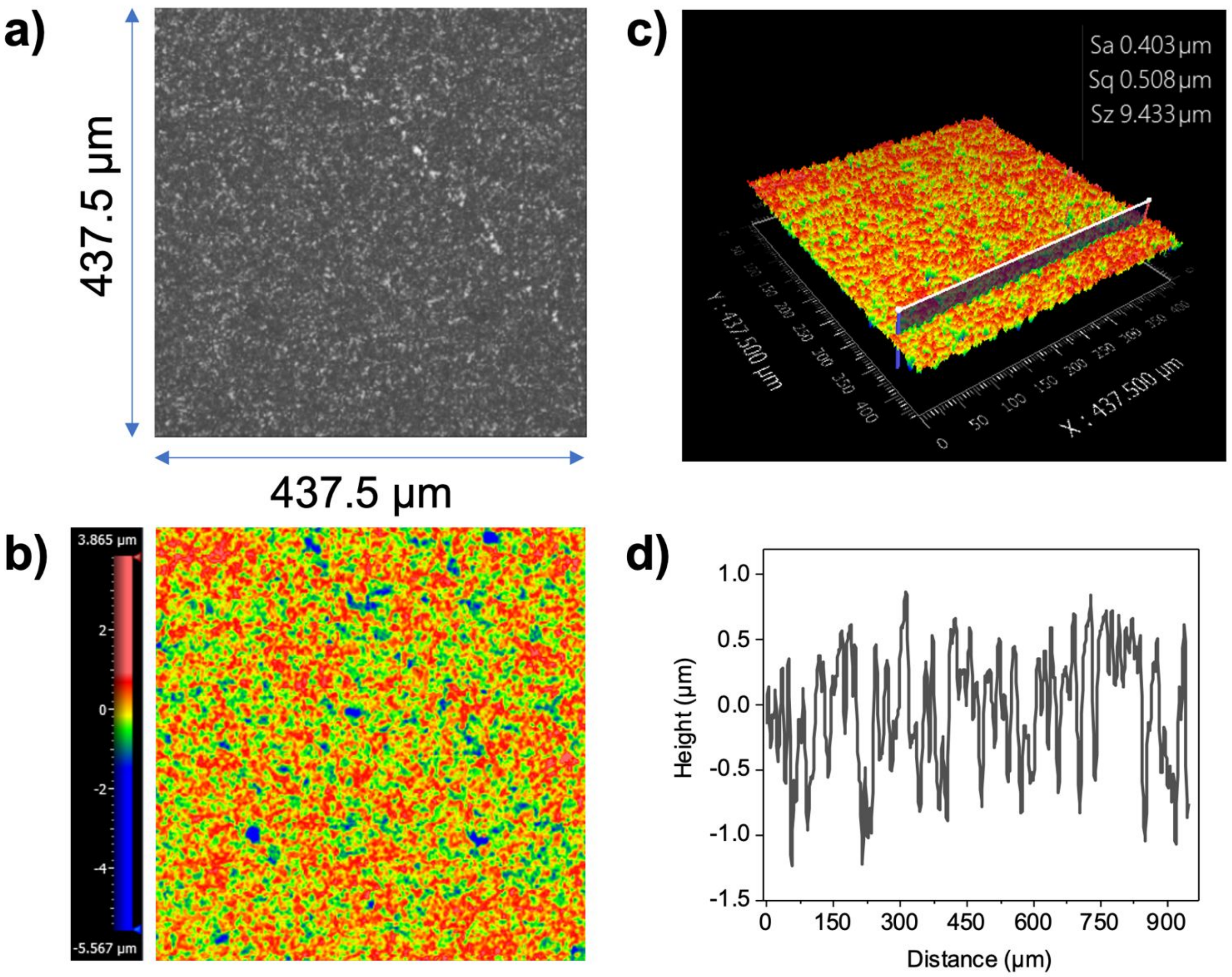


**Figure S12.** Surface analysis of the sample after polished by diamond grinding disc (MD 120). a) Zygo optical image of the location for surface topography analysis. b) Zygo 2-dimensional surface topography image of the location. c) Zygo 3-dimensional surface topography image of the location. d) The change of the surface topography in the target area along the slice.

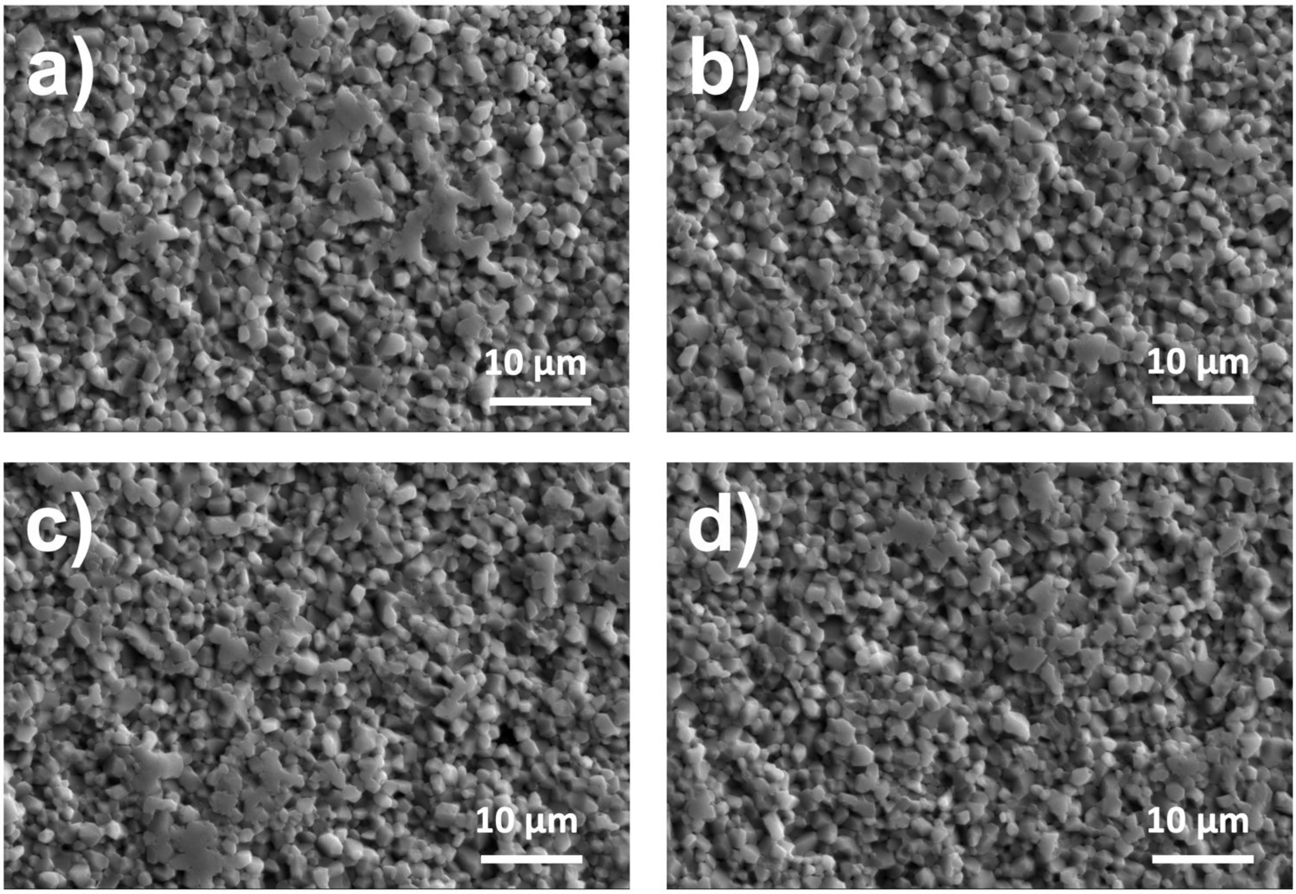


**Figure S13.** a) SEM image of the grinded substrate's surface structure with a grinding speed of 40 r/min. b) SEM image of the grinded substrate's surface structure with a grinding speed of 120 r/min. c) SEM image of the grinded substrate's surface structure with a grinding speed of 200 r/min. d) SEM image of the grinded substrate's surface structure with a grinding speed of 320 r/min.

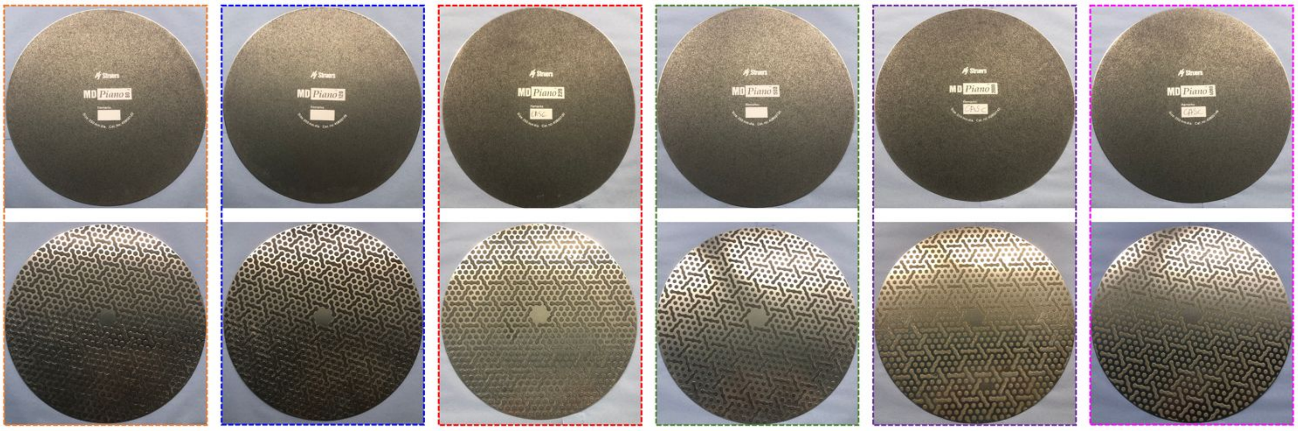

**Figure S14.** Photographs of diamond grinding discs which are comparable to the SiC sandpaper with grits of 80 (grinding particle size: 201 μm), 120 (grinding particle size: 137 μm), 220 (grinding particle size: 77 μm), 500 (grinding particle size: 37 μm), 2000 (grinding particle size: 10 μm), and 4000 (grinding particle size: 5 μm).

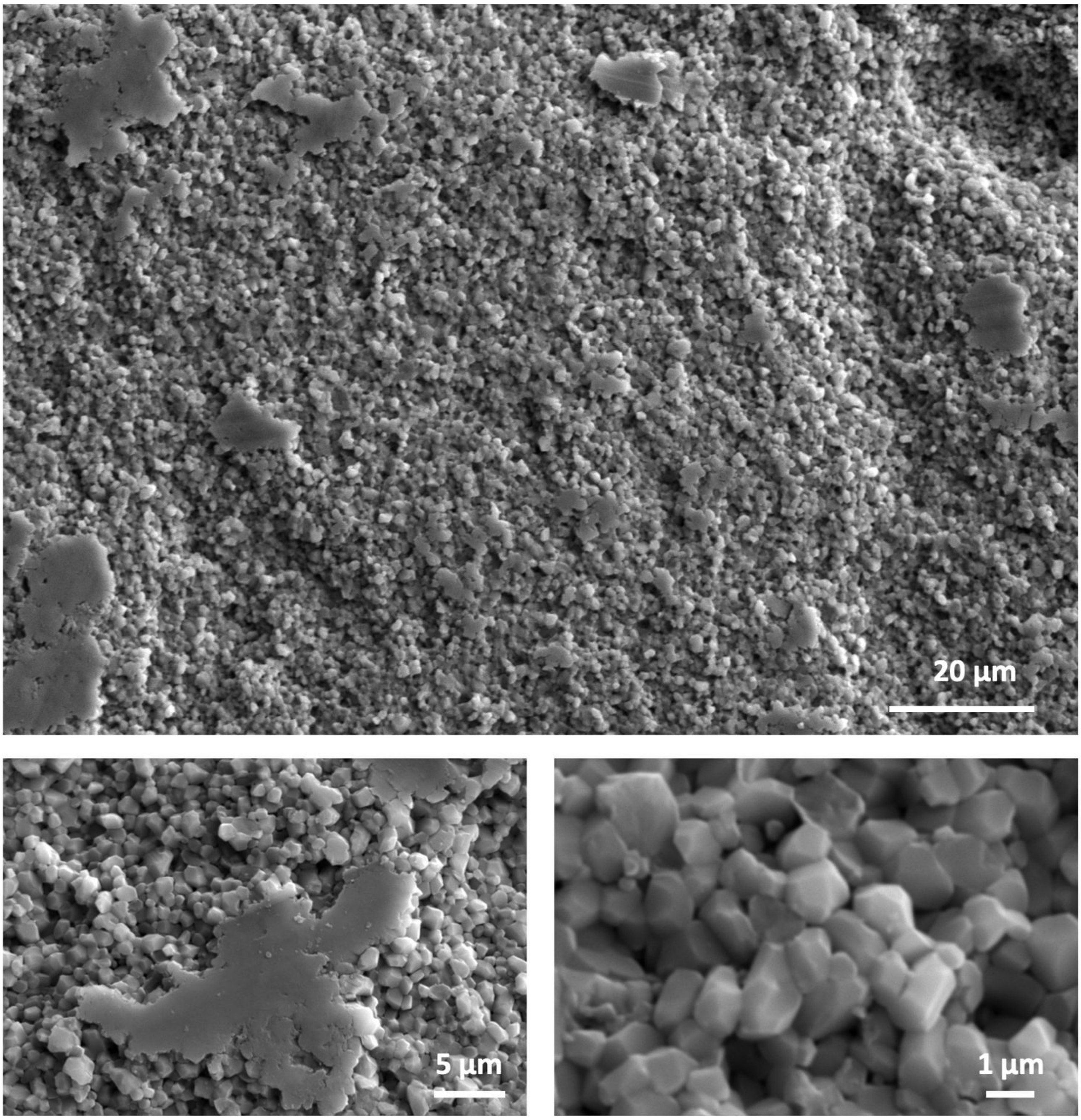


**Figure S15.** SEM image of the grinded substrate's surface structure.

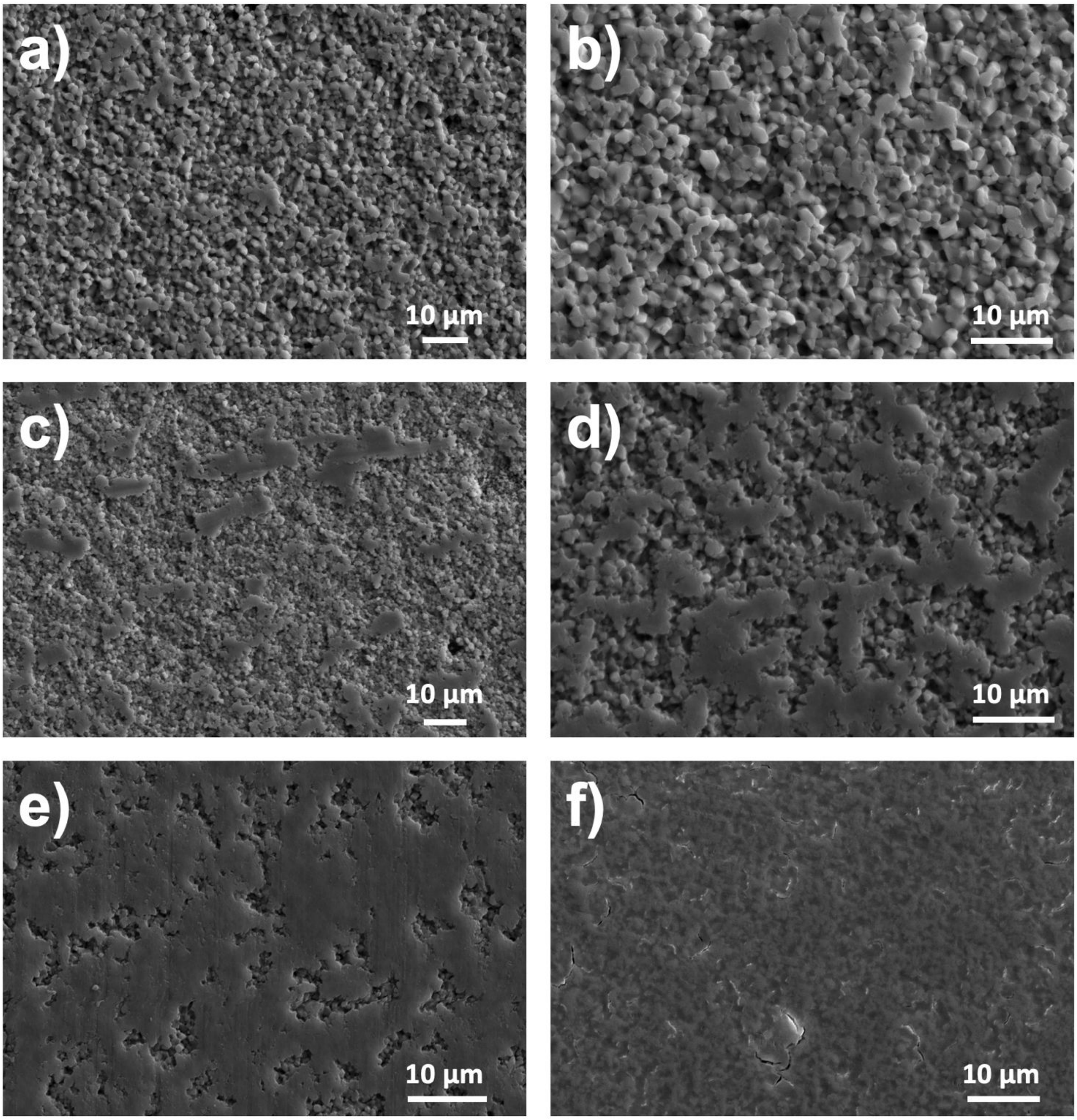


**Figure S16.** SEM image of the grinded substrate's surface structure grinded by different diamond discs which are comparable to the SiC sandpaper with grits of a) 80, b) 120, c) 220, d) 500, e) 2000 and f) 4000.

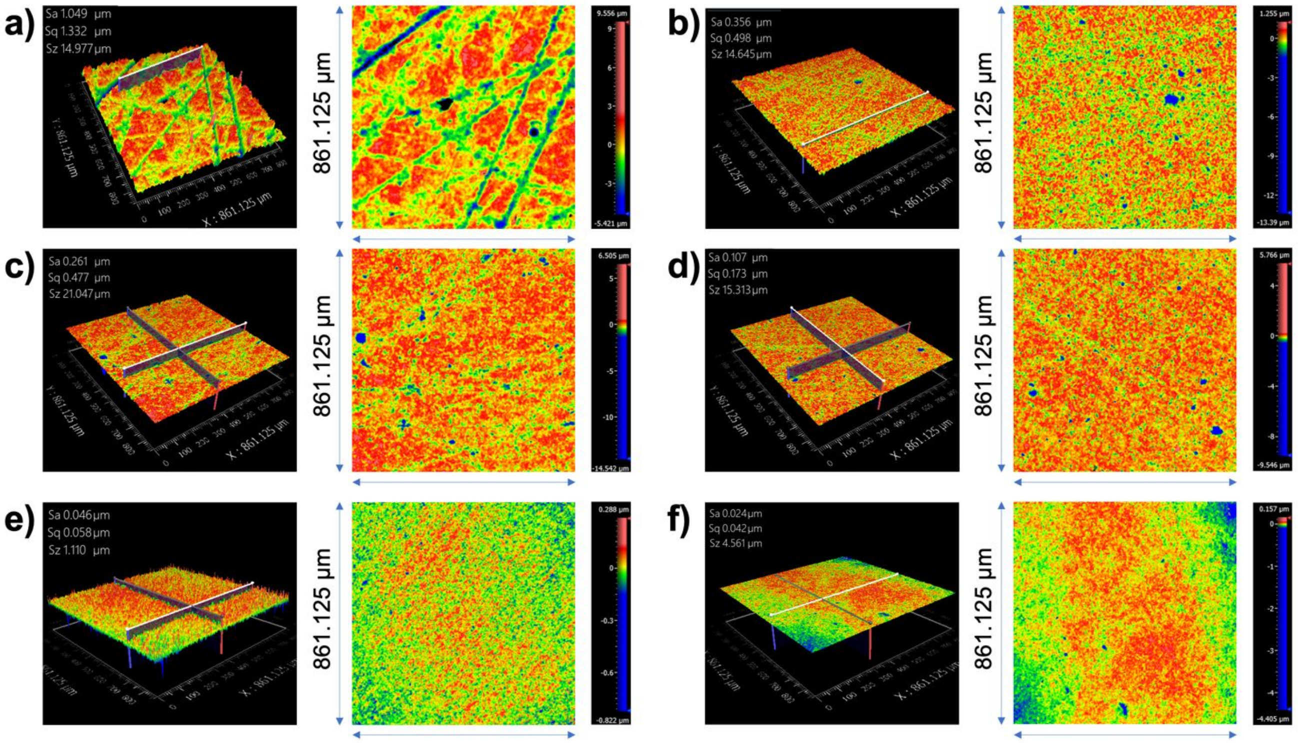


**Figure S17.** Zygo surface morphology image of the grinded substrate's surface structure grinded by different diamond discs which are comparable to the SiC sandpaper with grits of a) 80, b) 120, c) 220, d) 500, e) 2000 and f) 4000.

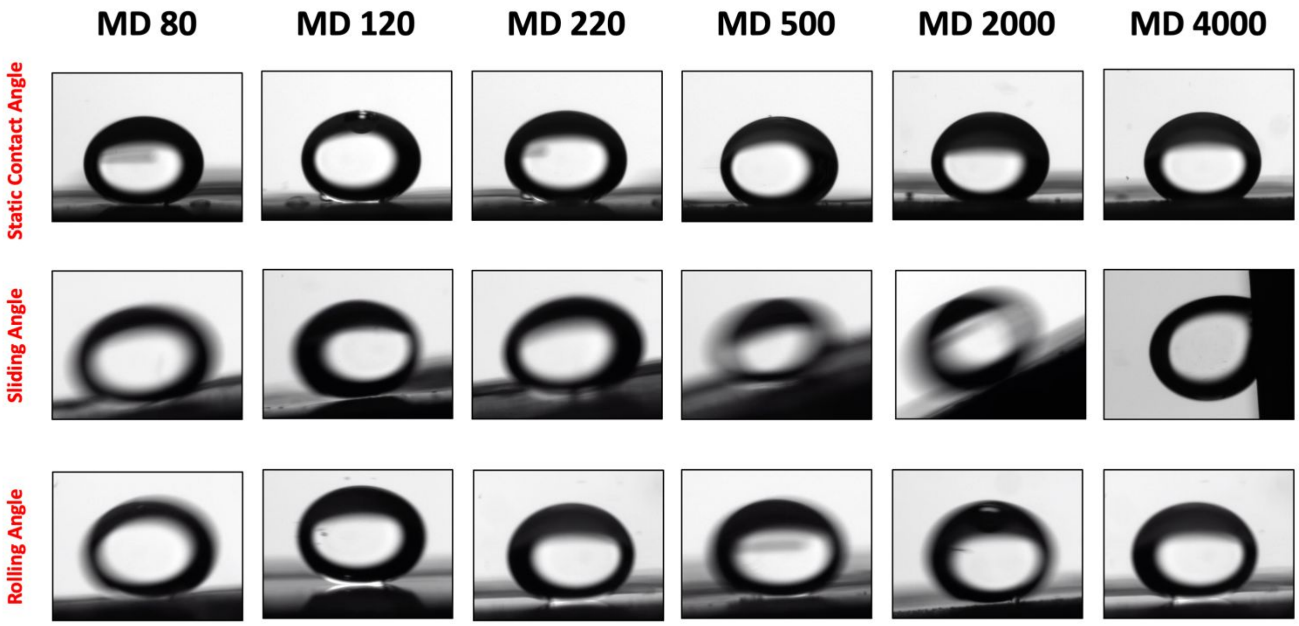


**Figure S18.** Static contact angles, sliding angles and rolling angles of the grinded substrates grinded by diamond with different grinding particle sizes. The applied oil was 1,2-dichloroethane (3 μL).

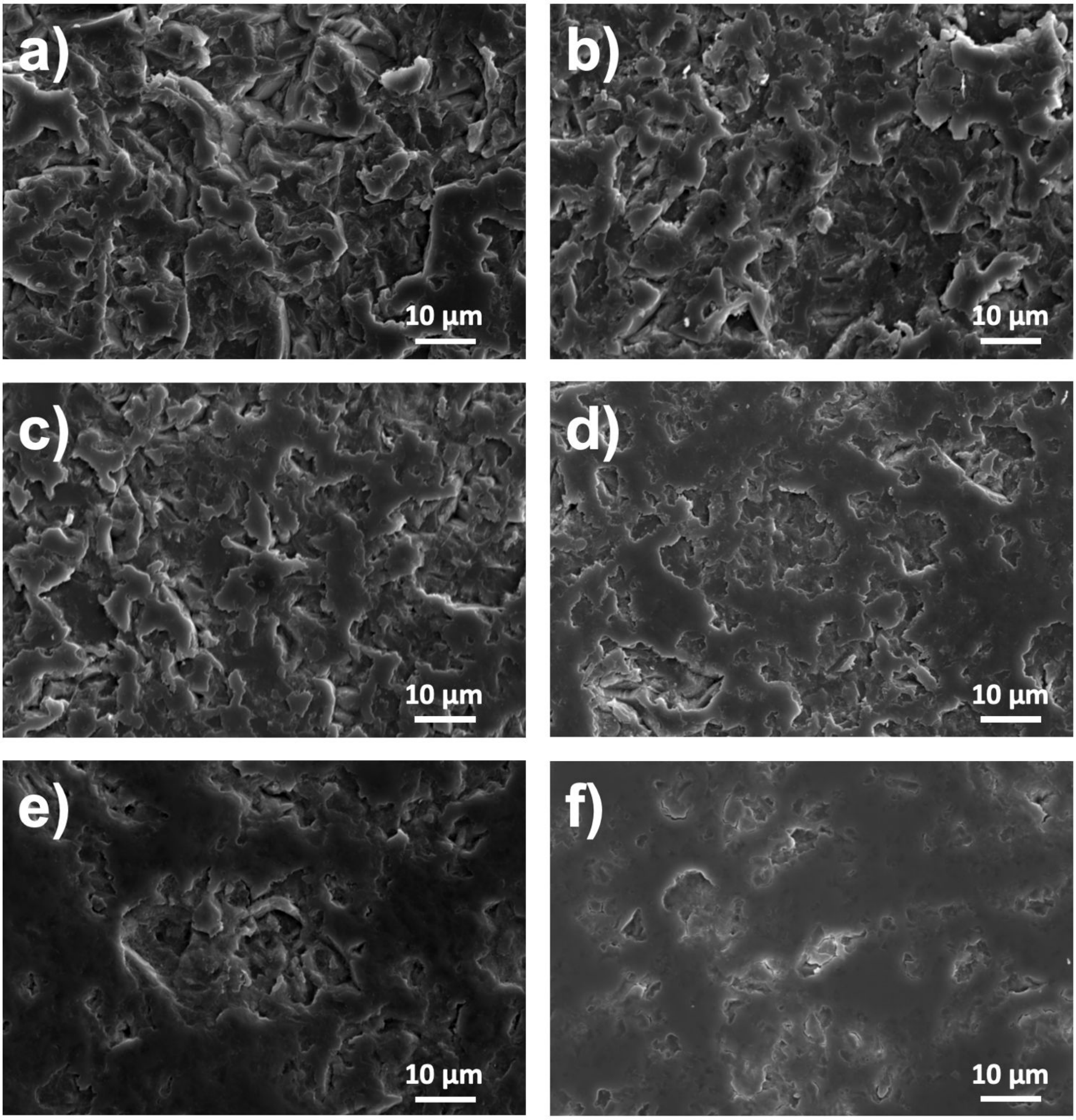


**Figure S19.** SEM image of the grinded spphires' surface structure grinded by different diamond discs which are comparable to the SiC sandpaper with grits of a) 80, b) 120, c) 220, d) 500, e) 2000 and f) 4000.

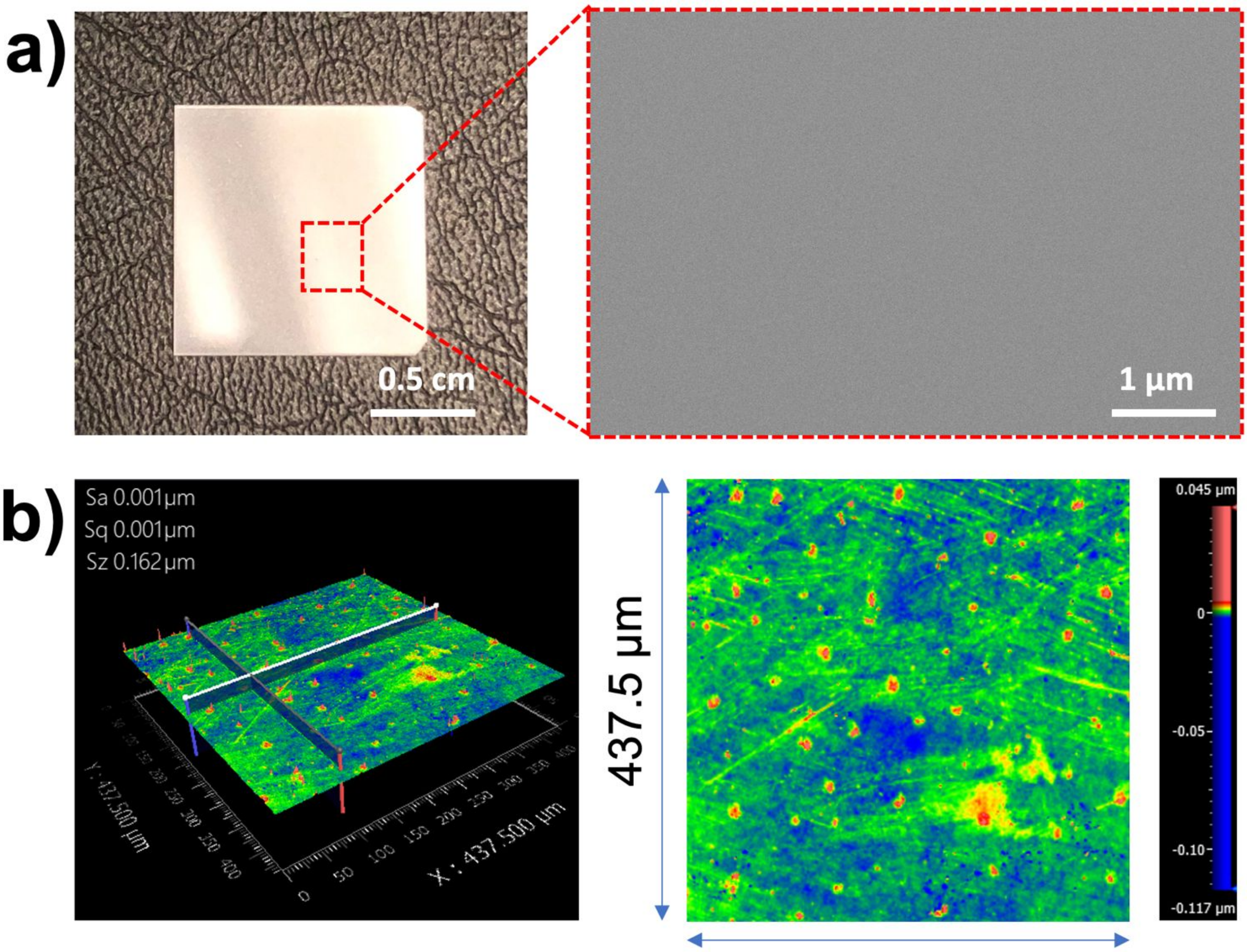


**Figure S20.** a) Photograph and SEM image of the surface structure of sapphire, showing a flat surface. b) Zygo surface morphology image of the sapphire surface.

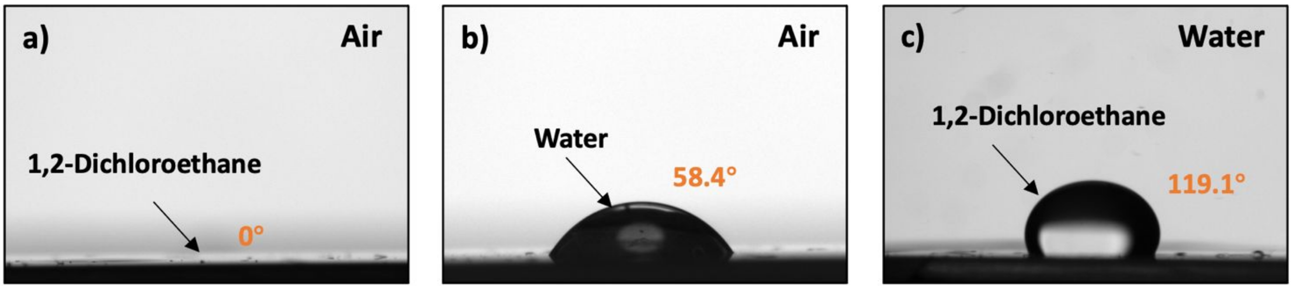


**Figure S21.** CAs on the sapphire: a) oil droplet on sapphire in air, b) water on sapphire in air, and c) oil droplet on sapphire in water. The applied oil was 1,2-dichloroethane (3 μL).

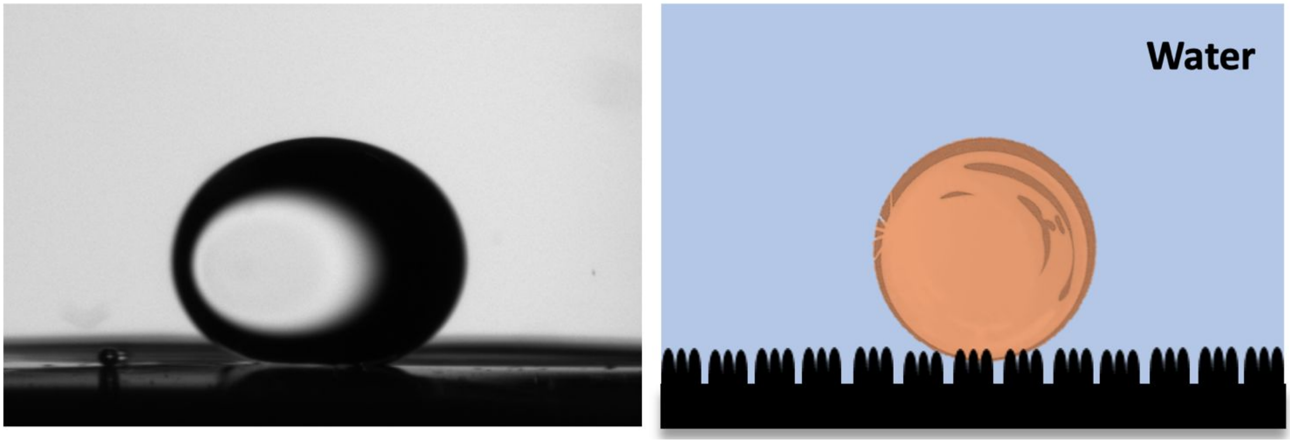


**Figure S22.** The contact situation between 1,2-dichloroethane (3 μL) and $Al_2O_3$ substrate in water.

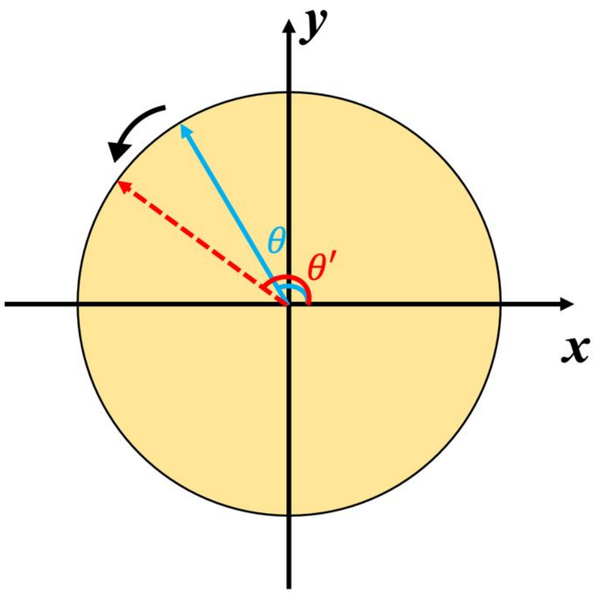


**Figure S23.** Schematic diagram of the change of the underwater contact angle between the oil droplet and the ceramic surface when the ceramic surface roughness increases.

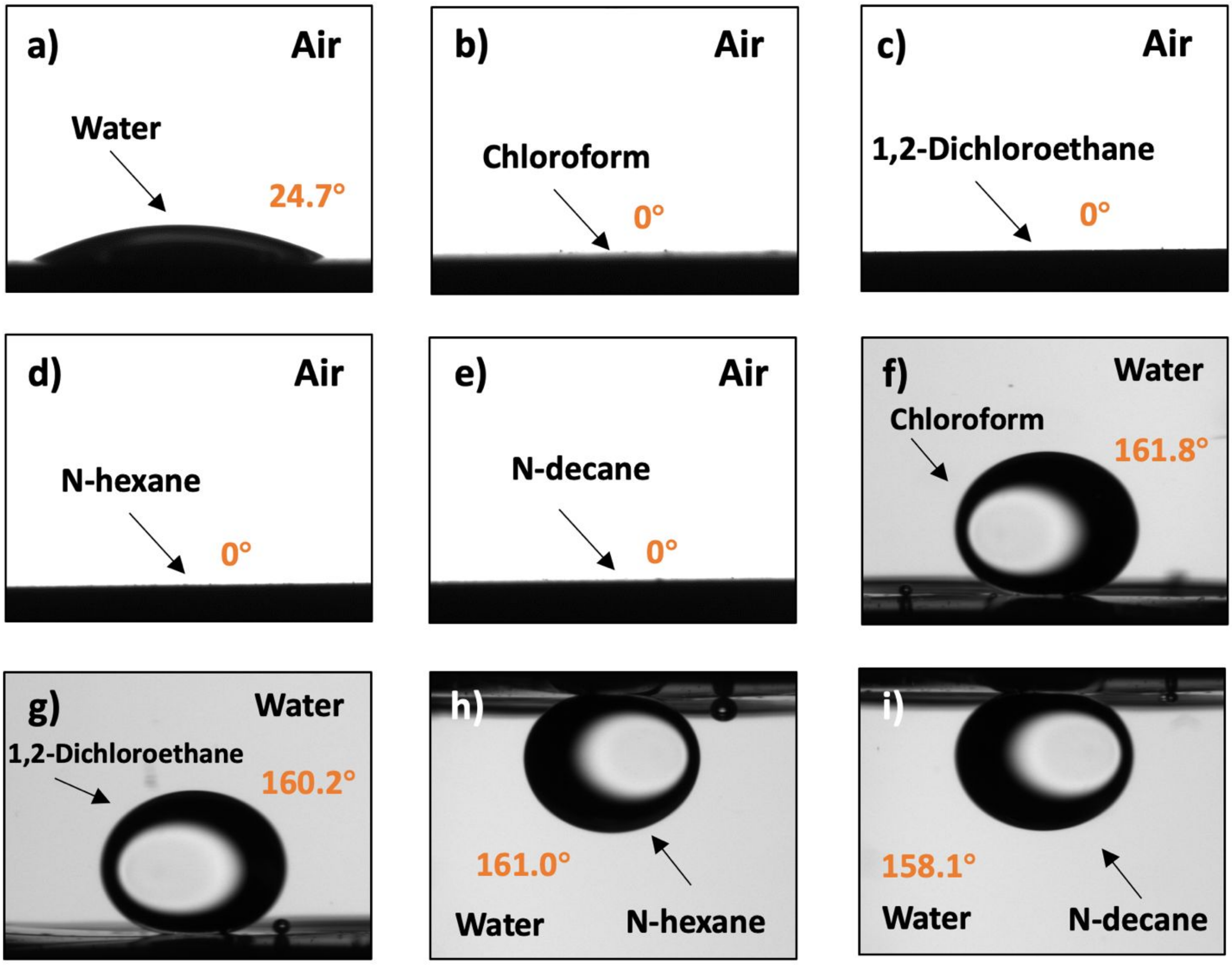


**Figure S24.** Contact angles on the sintered $Al_2O_3$ substrate: a) water in air, b) chloroform in air, c) 1,2-dichloroethane in air, d) N-hexane in air, e) N-decane in air, f) chloroform in water, g) 1,2-dichloroethane in water, h) N-hexane in water, and i) N-decane in water. The as-prepared $Al_2O_3$ substrate exhibits hydrophilic and lipophilic properties in the air, and super-oleophobic properties in water. The volume of all oil droplets were 3 μL.

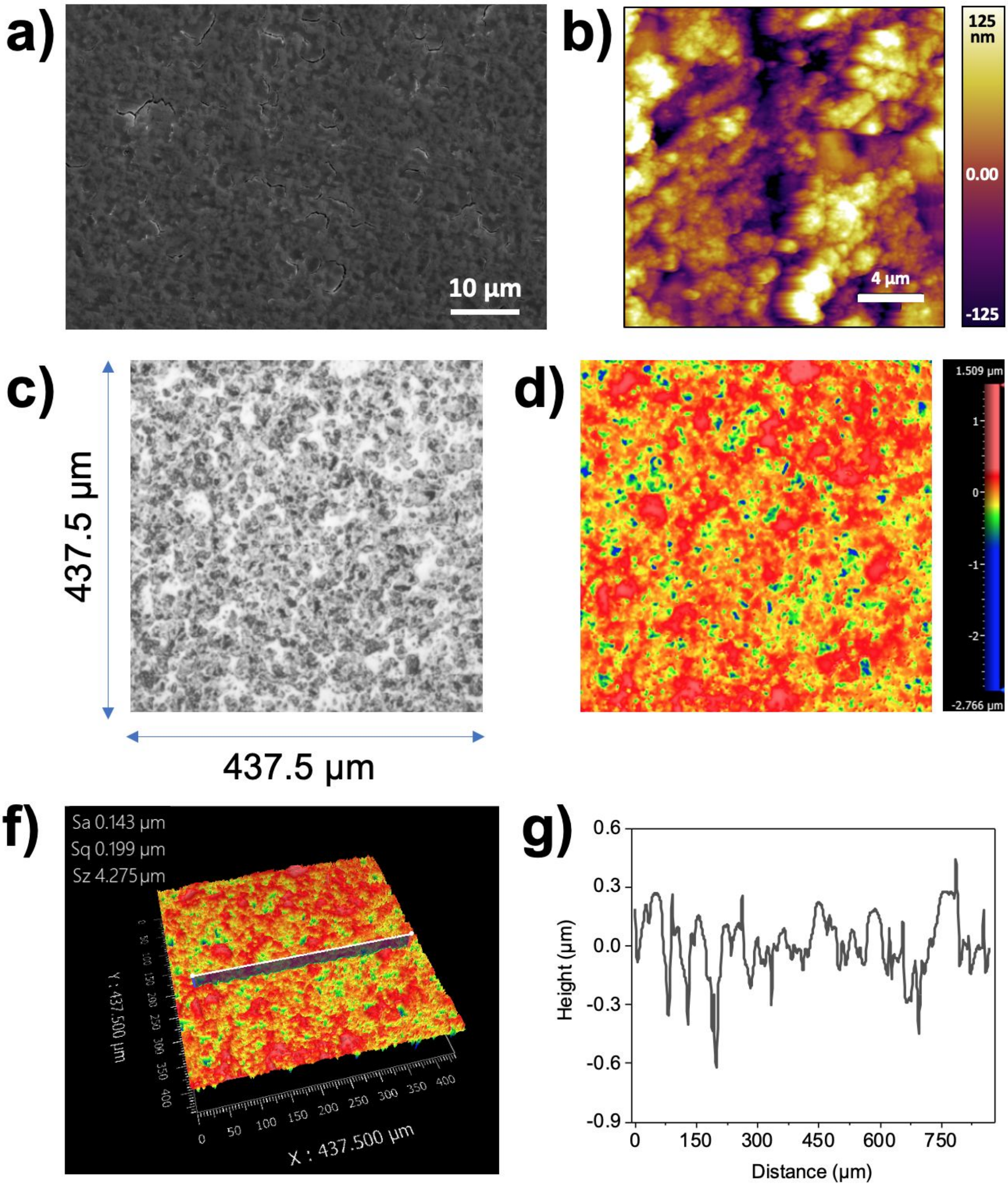


**Figure S25.** Surface analysis of the sample after polished by diamond grinding disc (MD 4000). a) SEM image of the grinded substrate's surface structure. b) AFM image of the grinded substrate's surface structure. c) Zygo optical image of the location for surface topography analysis. d) Zygo 2-dimensional surface topography image of the location. e) Zygo 3-dimensional surface topography image of the location. f) The change of the surface topography in the target area along the slice.

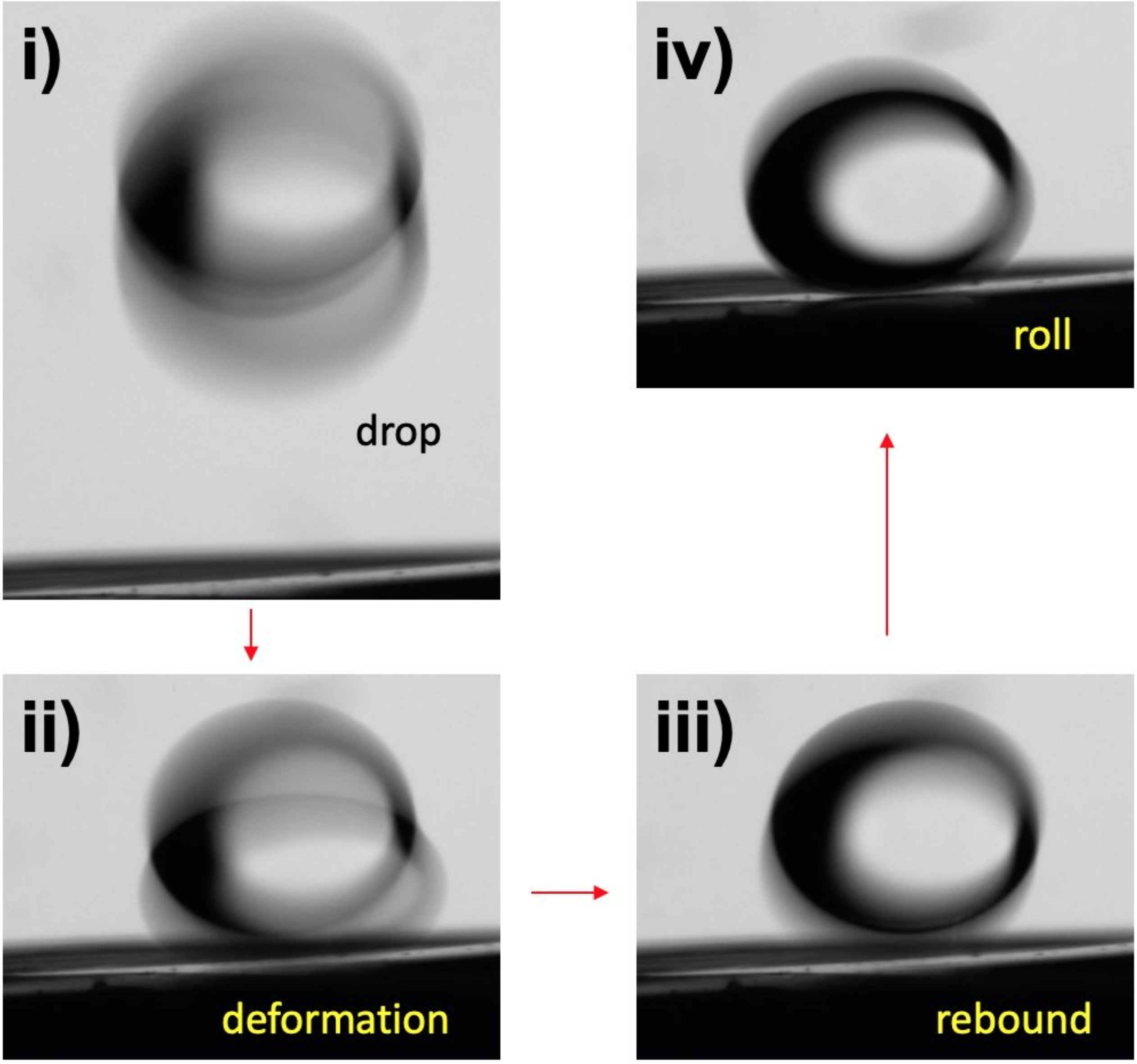


**Figure S26.** The state change process of the oil droplets during the rolling angle test. The applied oil was 1,2-dichloroethane (3 μL).

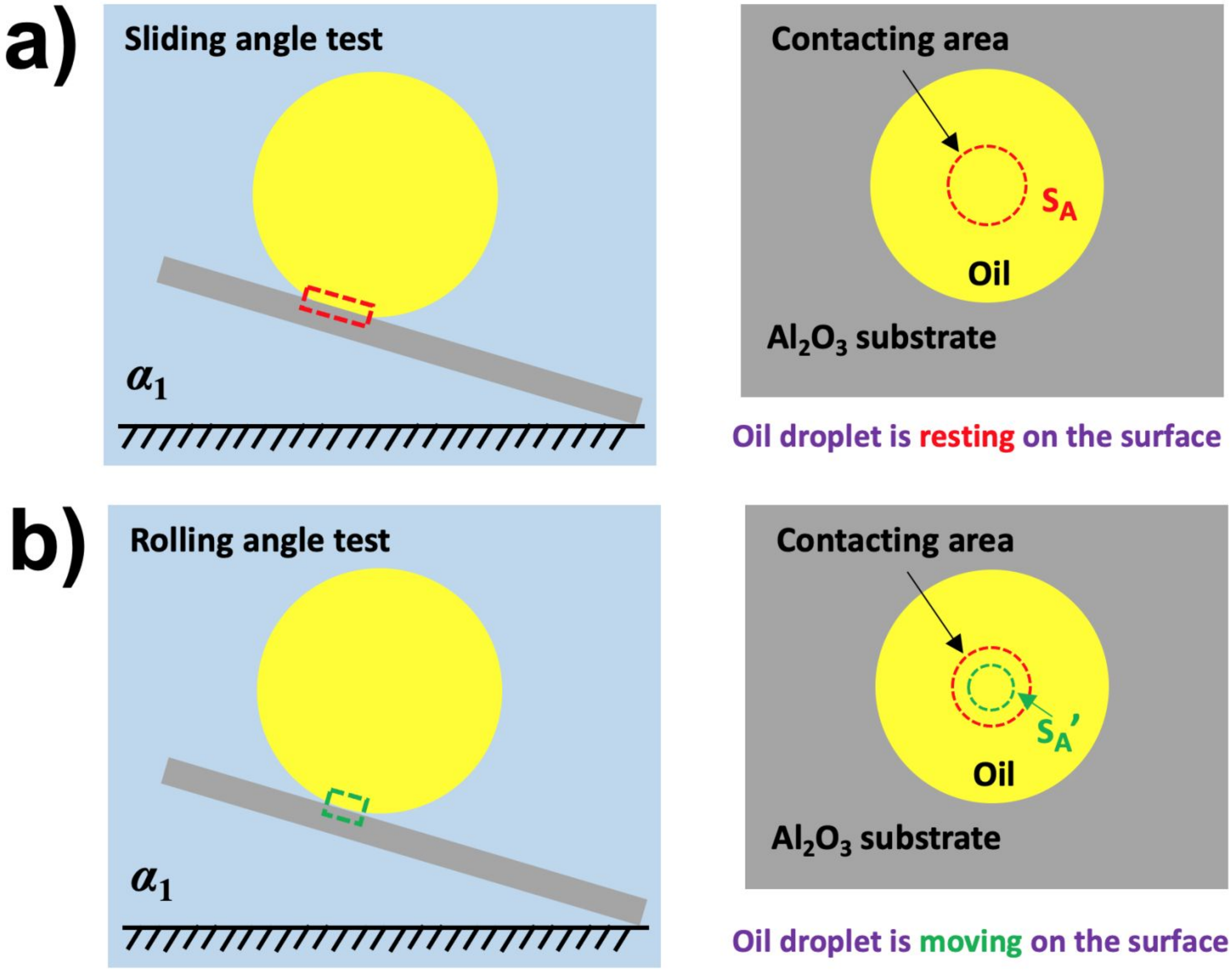


**Figure S27.** Schematic diagram of the actual contacting area between the oil droplet and the substrate. a) sliding angle test, b) rolling angle test.

## Reference:


1. Guo, T.; Heng, L.; Wang, M.; Wang, J.; Jiang, L., Robust Underwater Oil-Repellent Material Inspired by Columnar Nacre. *Advanced Materials* **2016,** *28* (38), 8505-8510.

2. Meng, X.; Wang, M.; Heng, L.; Jiang, L., Underwater Mechanically Robust Oil-Repellent Materials: Combining Conflicting Properties Using a Heterostructure. *Advanced Materials* **2018,** *30* (11), 1706634.

3. Li, C.; Lai, H.; Cheng, Z.; Yan, J.; Xiao, L.; Jiang, L.; An, M., Coating “Nano-Armor” for Robust Superwetting Micro/Nanostructure. *Chemical Engineering Journal* **2020,** *385*, 123924.

4. Chen, W.; Zhang, P.; Zang, R.; Fan, J.; Wang, S.; Wang, B.; Meng, J., Nacre-Inspired Mineralized Films with High Transparency and Mechanically Robust Underwater Superoleophobicity. *Advanced Materials* **2020,** *32* (11), 1907413.
